# Spin-dependent reactivity and spin-flipping dynamics in oxygen atom scattering from graphite

**DOI:** 10.1038/s41557-023-01204-2

**Published:** 2023-05-22

**Authors:** Zibo Zhao, Yingqi Wang, Ximei Yang, Jiamei Quan, Bastian C. Krüger, Paula Stoicescu, Reed Nieman, Daniel J. Auerbach, Alec M. Wodtke, Hua Guo, G. Barratt Park

**Affiliations:** 1 Max-Planck-Institut für Multidisziplinäre Naturwissenschaften, Göttingen, Germany; 2 Department of Chemistry and Chemical Biology, University of New Mexico, Albuquerque, NM USA; 3 Georg-August-Universität Göttingen, Institut für physikalische Chemie, Göttingen, Germany; 4 International Center for Advanced Studies of Energy Conversion, University of Goettingen, Göttingen, Germany; 5 Department of Chemistry and Biochemistry, Texas Tech University, Lubbock, TX USA

**Keywords:** Physical chemistry, Surface chemistry, Molecular dynamics, Excited states

## Abstract

The formation of two-electron chemical bonds requires the alignment of spins. Hence, it is well established for gas-phase reactions that changing a molecule’s electronic spin state can dramatically alter its reactivity. For reactions occurring at surfaces, which are of great interest during, among other processes, heterogeneous catalysis, there is an absence of definitive state-to-state experiments capable of observing spin conservation and therefore the role of electronic spin in surface chemistry remains controversial. Here we use an incoming/outgoing correlation ion imaging technique to perform scattering experiments for O(^3^P) and O(^1^D) atoms colliding with a graphite surface, in which the initial spin-state distribution is controlled and the final spin states determined. We demonstrate that O(^1^D) is more reactive with graphite than O(^3^P). We also identify electronically nonadiabatic pathways whereby incident O(^1^D) is quenched to O(^3^P), which departs from the surface. With the help of molecular dynamics simulations carried out on high-dimensional machine-learning-assisted first-principles potential energy surfaces, we obtain a mechanistic understanding for this system: spin-forbidden transitions do occur, but with low probabilities.

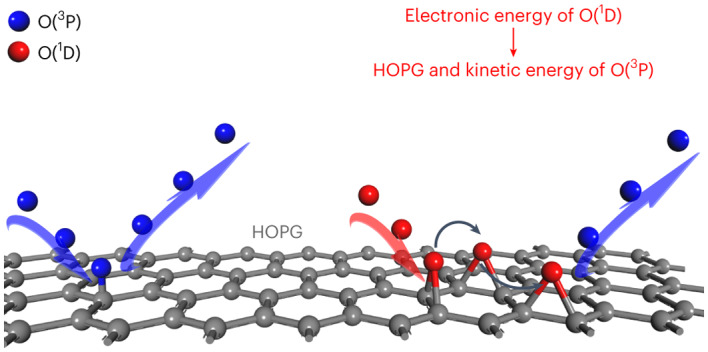

## Abstract (web summary)

Electronic spin influences chemistry profoundly, but its role in surface chemistry is poorly established. Now the spin-dependent reaction probabilities of oxygen atoms with a graphite surface have been studied. Molecular dynamics simulations help elucidate the mechanism for spin-flipping, which is observed to occur with low probability in surface scattering experiments.

## Main

The influence of electronic spin on the outcome of molecular collisions is of fundamental importance in understanding chemical reaction pathways and rates and often has dramatic effects^1^. In gas-phase reactions and photochemistry, electronic spin conservation leads to well-known selection rules. For example, O(^1^D) reacts without a barrier by inserting from the side into an H–H bond to form water^2^, whereas O(^3^P) reacts at one end of the molecule forming OH + H, which requires substantial activation^3^. On the other hand, spin-forbidden reactions are known, particularly for systems involving heavy atoms^4^. Spin–orbit couplings can be computed for simple gas-phase reactions^5^, and the role of spin in gas-phase reaction dynamics has become well understood.

In contrast, for surface reactions, so important to topics like catalysis, corrosion and microelectronic materials processing, spin-selective reactivity is controversial. For example, spin selection rules have been invoked to explain conflicting results coming from experiment and theory for the dissociative chemisorption of O_2_ (${{}^{{{\mathrm{3}}}}{{{\mathrm{{\Sigma}}}}}_{\rm{g}}^ {-}}$) on Al(111). Experiments show that the reaction becomes more probable when O_2_(${{}^{{{\mathrm{3}}}}{{{\mathrm{{\Sigma}}}}}_{\rm{g}}^ {-}}$) collides at the surface with high translational energy^6^, a clear indication of a barrier to dissociative adsorption. However, when density functional theory (DFT) calculations are performed at the level of generalized gradient approximation (GGA), no barrier is found^7,8^. One school of thought supposes that the reaction must occur with conservation of triplet spin, and, indeed, spin-restricted DFT calculations predict a barrier to reaction^7,8^. An alternative explanation involves charge transfer leading to transient ${{{{{\mathrm{O}}}}_{2}}^ {-}}$ formation, where barriers are found only when correlated wavefunction methods are used. In this line of reasoning, weaknesses in the ability of DFT-GGA to describe charge transfer are the source of inconsistency between experiment and theory^9,10^.

Although the work just described reveals important features of surface chemistry, especially the care that must be taken when attempting electronic structure calculations of reaction paths, they leave unanswered the questions of whether and under what conditions electronic spin is important in surface chemistry and when it is unlikely to be unimportant. For example, it has recently been demonstrated that the electrons’ spin degeneracy lowers the reaction-rate constant by fourfold in H–H recombination on metals, as has long been known for H–H recombination in the gas phase^11^. The fate of atomic spin polarization in H scattering from surfaces has been explored theoretically^12^, but, to the best of our knowledge, there are no experiments addressing spin-dependent reactivity and/or spin-flipping pathways.

The reaction of graphitic carbon with atomic oxygen has been the subject of great interest^13–22^ due to its importance in the degradation of aerospace materials^21^ and the use of oxygen plasma etching in nanoscale materials and device fabrication^23–26^. Electronically nonadiabatic dynamics have been considered for O-atom scattering from graphite by application of ab initio direct Ehrenfest dynamics^27^. In this Article, we report spin-resolved state-to-state scattering of O atoms from a highly oriented pyrolytic graphite (HOPG) surface, using a novel ion imaging technique to measure reaction probabilities and differential scattering cross-sections with high velocity resolution for both incident and scattered atoms, even when the incident atomic beam has a broad velocity distribution. We analyse these experimental observations with the help of novel spin-state selective molecular dynamics (MD) simulations using newly developed high-dimensional potential energy surfaces (PESs), machine-learned from DFT data. The results clearly demonstrate that O(^1^D) undergoes much more efficient reaction with HOPG than does O(^3^P), exhibiting higher sticking probabilities than its high-spin counterpart. We also observe electronically nonadiabatic pathways, where O(^1^D) quenches to O(^3^P), releasing excess kinetic energy. The mechanism of quenching involves a spin flip—singlet to triplet—of a highly vibrationally excited chemisorbed O atom formed from incident O(^1^D) that takes place before it has equilibrated fully with the surface.

## Results

### Calculated PESs

**Fig. 1:**
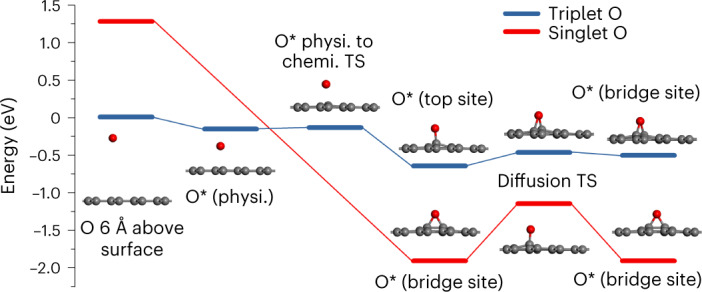
DFT stationary points on the triplet and singlet PESs for atomic oxygen on graphene. The triplet PES (blue) has a shallow physisorption (physi.) well with O 2.3 Å above the surface and exhibits chemisorption (chemi.) at the top and bridge sites with binding energies of 0.65 and 0.51 eV, respectively. The singlet PES (red) does not have a physisorption well but exhibits strong chemisorption at the bridge site (1.91 eV binding energy relative to the O(^3^P) asymptote). The two PESs cross at a seam of intersection located near the physisorption barrier on the triplet PES. Note that, due to intrinsic errors in the DFT calculations, the calculated gas-phase splitting between the O(^1^D) and O(^3^P) states is only 1.3 eV, whereas experimentally this quantity is known to be 1.97 eV. Therefore, the singlet PES was shifted upwards by 0.7 eV in the O(^1^D) scattering simulations to match the ^1^D/^3^P energy gap of the atomic oxygen. TS, transition state. Source data

The lowest-lying triplet and singlet PESs for O on graphene were calculated using spin-constrained DFT. The calculated surface binding configurations and saddle points are compared in Fig. 1. The triplet PES has a shallow physisorption well ~2.3 Å above graphene and exhibits stable chemisorption at the top and bridge sites with binding energies of 0.65 and 0.51 eV, respectively. On the singlet PES, no physisorption state was found. The bridge site is the only stable binding site, forming an epoxide moiety with an elongated C–C bond (*r* = 1.5 Å) and two C–O bonds (*r* = 1.5 Å). The binding energy at the bridge site is 1.91 eV relative to the triplet asymptote. These results are consistent with previous DFT calculations^14,18,19,22,28^, which follow the lower adiabat because the spin of the system was not specified. The PESs of the two spin states cross when the O atom is relatively far (~2 Å) from the surface and the intersection seam is almost isoenergetic to the triplet asymptote (Supplementary Fig. 9), but the crossing seam might contain fairly large uncertainties given the inability of DFT to reproduce the ^1^D-^3^P energy difference of atomic oxygen. For the impinging O(^3^P) atom, it is possible to hop to the singlet state or stay in the triplet state, which will lead to different dynamical results. The incident O(^1^D) atoms also face the choice of either staying on the singlet state or hopping to the triplet state as they reach the intersection.

### Scattering of O(^3^P)

**Fig. 2:**
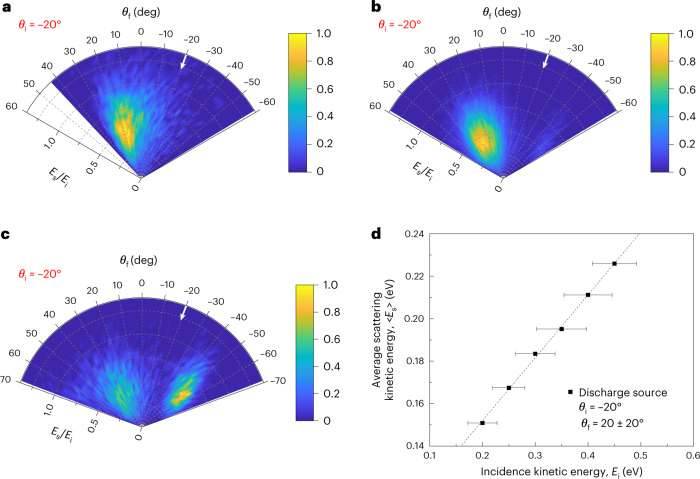
Experimental and theoretical scattering angle and kinetic energy distributions for O(^3^P) → O(^3^P) scattering. **a**, Discharge source. The experimental kinetic energy and angular distribution of O(^3^P_2_), obtained after scattering atomic O (*E*_i_ = 0.34 ± 0.05 eV) from HOPG at an incidence angle *θ*_i_ of −20°. **b**, Triplet PES. The calculated scattering distribution obtained using the triplet PES with an incidence energy of *E*_i_ = 0.34 eV and *θ*_i_ of −20°. **c**, Adiabatic PES. The calculated scattering distribution obtained using the adiabatic PES (*E*_i_ = 0.34 eV, *θ*_i_ = −20°). The experimental distribution (**a**) is consistent with the calculated distribution obtained using the triplet PES (**b**), but differs from the distribution obtained using the adiabatic PES (**c**), suggesting that spin transitions are not likely to occur during direct scattering trajectories. **d**, The average scattered kinetic energy, derived from experiment, is plotted as a function of incidence kinetic energy. The horizontal error bars indicate the FWHM of the incidence energy distribution corresponding to each data point. Source data

Two methods are used to generate atomic oxygen beams with different initial O-atom spin-state distributions: photolysis, which favours the formation of O(^1^D), and electric discharge, which favours O(^3^P). Using the discharge source (containing 87% O(^3^P)), we measured the energy and angular distributions of scattered O atoms. We searched for atoms in the O(^1^D) state by tuning the laser to the appropriate wavelength, but did not detect any scattered O(^1^D) signal. Using the incoming/outgoing ion imaging correlation method, we determined the angular and kinetic energy distributions of scattered O(^3^P) atoms with <*E*_i_> = 0.34 ± 0.05 eV at an incidence angle of −20° (*E*_i_, incidence energy). The results are displayed in Fig. 2a. (Distributions obtained at other incidence angles are presented in Extended Data Fig. 2). The angular distribution peaks close to the specular angle, and the energy distribution peaks at a value of *E*_s_/*E*_i_ ≈ 0.47 (*E*_s_, scattering energy). Furthermore, the average scattering energy, integrated over all scattering angles, clearly depends on the incidence energy, as shown in Fig. 2d. These results indicate that the observations are dominated by a direct inelastic scattering mechanism with sub-picosecond surface residence time, as has been reported previously in O/HOPG scattering experiments performed at a surface temperature of *T*_surf_ = 298 K (refs. ^21,29^).

### O(^1^D) scattering experiments

**Fig. 3:**
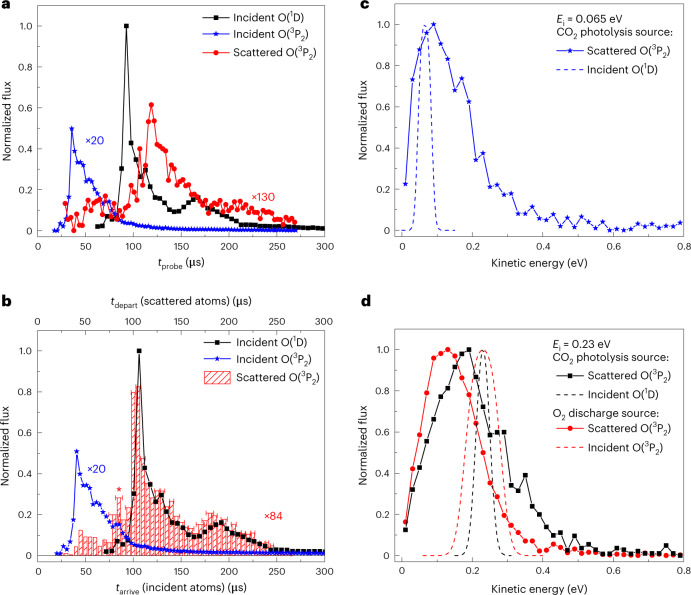
Scattering of O(^1^D) from HOPG leads to a spin non-conserving O(^1^D) → O(^3^P) channel that exhibits electronic-to-kinetic energy transfer. **a**, The flux of incident O(^3^P_2_) (blue stars), incident O(^1^D) (black squares) and scattered O(^3^P_2_) (red circles) in the CO_2_ photolysis experiment, plotted as a function of *t*_probe_, the time between the photolysis laser and probe laser. **b**, The *t*_arrive_ distribution for incident O(^1^D) and O(^3^P_2_) is compared with the *t*_depart_ distribution for scattered O(^3^P_2_). The widths of the red shaded rectangles indicate the size of the *t*_depart_ histogram intervals and the horizontal error bars indicate the FWHM uncertainty in *t*_depart_ due to uncertainty in the velocity measurements. The peak at 85 μs (marked with an asterisk) is an artefact due to a small contamination of O_2_ in the CO_2_ molecular beam. A comparison of the profile of the *t*_depart_ distribution with the *t*_arrive_ distributions indicates that incident O(^1^D) atoms are converted to O(^3^P) during the scattering process. **c**, The scattering kinetic energy distribution for the O(^1^D) → O(^3^P_2_) channel is shown for incident O assigned to *E*_i_ = 0.065 ± 0.020 eV (blue stars). The distribution of incidence energies (including experimental uncertainty) is shown as a blue dashed curve. Atoms gain kinetic energy during the scattering process due to electronic-to-kinetic energy coupling. **d**, The scattered O(^1^D) → O(^3^P_2_) kinetic energy distribution from the CO_2_ photolysis source assigned to *E*_i_ = 0.23 ± 0.02 eV (black squares) is compared to the distribution obtained from the O_2_ discharge source (red circles) with a similar range of incidence energies (*E*_i_ = 0.23 ± 0.05 eV), indicating that, on average, incident O(^1^D) atoms scatter with higher kinetic energy than incident O(^3^P) atoms with similar incidence kinetic energy. The incidence energy distributions are indicated by black and red dashed curves, respectively. Source data

The 157-nm photolysis of CO_2_ was used to obtain an incident beam composed of 94% O(^1^D) and 6% O(^3^P). The O(^3^P) atoms are faster and consequently arrive at the surface much earlier than the O(^1^D) atoms. Figure 3a shows the flux of incident and scattered O atoms as a function of *t*_probe_. Incident O(^3^P_2_) atoms reach the detection laser ~40 μs after the photolysis laser, whereas incident O(^1^D) atoms arrive between ~80 and 220 μs after. Note that the surface arrival time distributions of the O(^1^D) and O(^3^P) atoms are separated in time, making state-to-state measurements of the scattered atom distribution possible. No scattered atoms could be detected in the O(^1^D) state, but scattered O(^3^P) atoms were detected with a broad *t*_probe_ distribution between 30 and 250 μs after the photolysis laser, as also shown in Fig. 3a.

Figure 3b shows the results when applying the incoming/outgoing correlation method (Methods). Here we compare the distribution of *t*_arrive_ for the incident O(^1^D) and O(^3^P) atoms with the distribution of *t*_depart_ for the scattered O(^3^P) atoms. (The details of the correction for the surface to probe laser flight time are illustrated in Supplementary Fig. 3). There is a small contribution of incident O(^3^P) atoms at early times, giving rise to a peak at ~40 μs, but the primary scattering component, which extends from 90‒250 μs, has a *t*_depart_ distribution that closely matches the surface-arrival time distribution of O(^1^D) atoms, which allows us to definitively assign the primary component of the signal from O(^3^P) leaving the surface to a spin-changing process involving O(^1^D) → O(^3^P) conversion induced by the collision at the surface. (Note that the small peak at 85 μs marked with an asterisk is an artefact arising from a small contamination of O_2_ in the CO_2_ precursor beam.)

Figure 3c shows the O(^1^D) → O(^3^P) scattering energy distribution for *E*_i_ = 0.065 ± 0.020 eV. The most probable scattering energy, ${E}_{{{\mathrm{s}}}}^{{{{\mathrm{MP}}}}}$, is ~0.1 eV, and the distribution extends more than 0.3 eV higher than *E*_i_. This excess translational energy must come from the incidence electronic energy (1.97 eV). Remarkably, only a small fraction (~5%) of the incidence electronic energy is converted to translation. The results also show that the spin-forbidden O(^1^D) → O(^3^P) channel occurs without complete thermalization of the adsorbed atom to the surface. Comparing Fig. 3c,d, where the incidence energy is increased from 0.065 to 0.23 eV, we see that ${E}_{{{\mathrm{s}}}}^{{{{\mathrm{MP}}}}}$ also increases from 0.1 to 0.2 eV. This memory effect rules out the possibility of a substantial residence time at the surface and at the same time validates the assumption *t*_arrive_ = *t*_depart_, used in the incoming/outgoing correlation method. Additional evidence of electronic to translational energy conversion is seen in Fig. 3d, where the scattering energy distributions obtained for incident O(^3^P) and O(^1^D) are compared at *E*_i_ = 0.23 ± 0.05 eV. Note that the observed energy of departing O(^3^P) is significantly smaller than that obtained for incident O(^1^D).

**Fig. 4:**
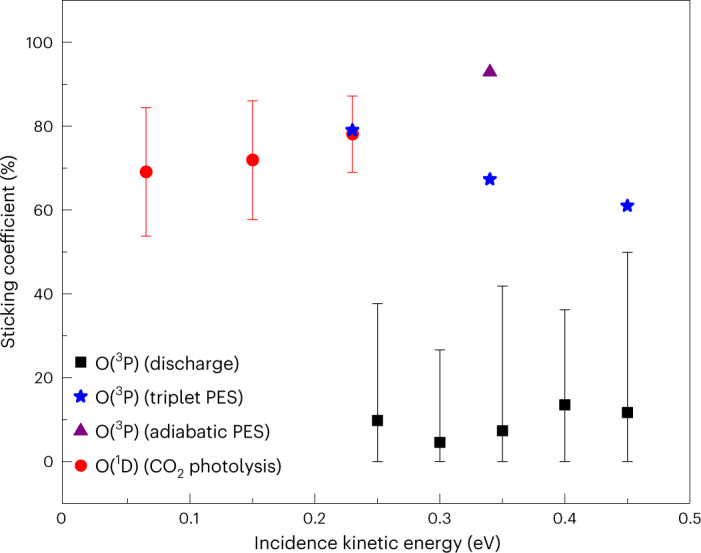
Comparison of the sticking coefficients of O(^3^P) and O(^1^D). Measured sticking coefficients for O(^1^D) from the CO_2_ photolysis experiment (red circles) and for O(^3^P) from the O_2_ discharge experiment (black squares) are plotted as a function of incidence kinetic energy. Data are represented as mean values, with error bars representing the 95% confidence intervals, obtained from a sample size of three repeated scattering measurements for O(^1^D) and five repeated measurements for O(^3^P). Calculated sticking coefficients for O(^3^P), obtained from trajectory calculations on the adiabatic PES (purple triangle) and triplet PES (blue stars), are also shown. Source data

The sticking probabilities of O(^1^D) are uniformly larger than those of O(^3^P), as shown in Fig. 4. Experimental sticking probabilities were derived by comparing the ratio of the total incident and scattered flux from the CO_2_ photolysis and O_2_ discharge sources, respectively, assigned via the incoming/outgoing correlation method (see Supplementary Section 2.4 for details). The incident O(^1^D) atoms that scatter from the surface undergo a spin transition and are detected as O(^3^P), as described earlier. Note that the incidence energy ranges of O(^1^D) (0.06‒0.23 eV) and O(^3^P) (0.25‒0.45 eV) in our experiment do not overlap and that the error bars are rather large. Nevertheless, it is clear that O(^1^D) at *E*_i_ = 0.23 eV exhibits ~8× higher sticking probability than O(^3^P) at almost the same energy (*E*_i_ = 0.25 eV).

### Simulation results for O(^3^P) scattering

To test the validity of spin conservation in the dynamics, MD simulations were carried out on both the triplet and adiabatic PESs. Figure 2 compares the experimental results (Fig. 2a), obtained for the O(^3^P) → O(^3^P) channel at *E*_i_ = 0.34 ± 0.05 eV and *θ*_i_ = −20°, with the theoretical results calculated for the same incidence conditions using both the triplet (Fig. 2b) and adiabatic (Fig. 2c) PESs. Similar results at different incidence angles are presented in Extended Data Fig. 2. The experimental distribution can only be reproduced by use of the triplet PES. Dynamics calculations on the adiabatic PES yield a bimodal angular distribution in which the backscattering peak can be attributed to the strong corrugation of the graphene surface towards the O atom. Detailed information about the surface corrugation and the backscattering mechanism is provided in Supplementary Section 2.3 (Extended Data Fig. 2 and Supplementary Figs. 8 and 9). No evidence of this backscattering peak is seen in the experiment. The sticking coefficients for O(^3^P) obtained from MD simulations are shown in Fig. 4, where the value from the spin-conserving (triplet) simulation is shown to be significantly smaller than that from the spin-relaxed adiabatic simulation and closer to the experiment. (Details are provided in Supplementary Section 2.5.) The calculated sticking coefficients are not in quantitative agreement with experiment—possibly due to the small sample size of the model—but we interpret the better overall agreement of the scattering distributions and sticking coefficients as evidence that O(^3^P) undergoes direct scatter on the triplet PES and not the adiabatic PES.

### Simulation results for O(^1^D) scattering

Two different MD simulations were performed for the scattering of O(^1^D) atoms at an incidence energy of 0.23 eV and an incidence angle along the surface normal, where comparison to experiment is possible. The first simulation employed the singlet PES, neglecting possible singlet–triplet crossing. Here, all trajectories led to sticking, which is consistent with the absence of O(^1^D) → O(^1^D) scattering in the experiment (Fig. 3b).

The second simulation models singlet–triplet coupling in a semi-empirical way. A proper simulation of the nonadiabatic intersystem crossing dynamics requires knowledge of the coordinate-dependent spin–orbit coupling between the singlet and triplet states, which is very challenging for this high-dimensional system and beyond the scope of this work. Instead, the following strategy was used to consider spin non-conserving dynamics.

As the impinging O(^1^D) atom first approaches the singlet–triplet seam, no transition to the triplet was permitted in our model; this is justified by the high velocity of the incident atom. The O atom then forms a hot adsorbate structure and begins relaxing to form the stable epoxide-like bridge site species. During the course of relaxation, the O atom continues to attempt crossings of the singlet–triplet seam, but it does so with decreasing velocity and thus increasing probability for a transition to the triplet. We simulated the effect of this time-varying spin-flip probability with the following ad hoc procedure. We imposed a singlet–triplet crossing delay, *τ*_S–T_. Any trajectory that reaches the singlet–triplet seam in the outgoing direction after this time delay is allowed to cross to the triplet PES with 100% probability. Very few such spin flips were observed, as the fast energy dissipation of the hot adsorbate rapidly prevents the trajectory from approaching the seam, which lies ~2 eV above the singlet PES minimum. See Supplementary Section 2.6 for more details.

**Fig. 5:**
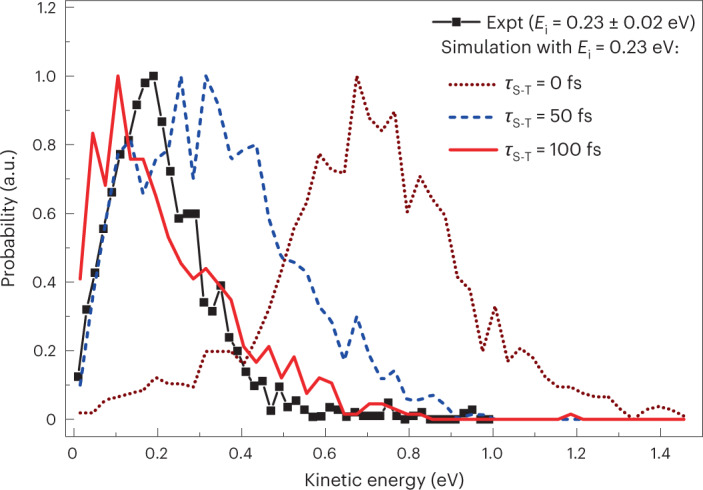
Final kinetic energy distribution for the scattered atomic oxygen, initially in the ^1^D state, from HOPG via the spin non-conserving O(^1^D) → O(^3^P) channel. Simulations were performed in which the spin flip was allowed to occur when the singlet/triplet seam of intersection is crossed in the outgoing direction only after a chosen delay, as described in the main text. Simulated results are shown for an incidence kinetic energy of 0.23 eV and delays of 0 fs (brown dotted line), 50 fs (blue dashed line) and 100 fs (red solid line). The experimental results corresponding to an incidence kinetic energy range of 0.23 ± 0.02 eV are shown for comparison (black squares). The experimental kinetic energy distribution is inconsistent with prompt O(^1^D) → O(^3^P) conversion at the seam of intersection (brown dotted line). Source data

Figure 5 compares the experimental kinetic energy distribution for scattered O(^3^P) atoms resulting from incident O(^1^D) with calculated results. When the singlet → triplet transition is assumed to occur immediately (*τ*_S–T_ = 0 fs), a much larger fraction of the electronic energy of incidence is channelled to translation energy of the scattered atom. As *τ*_S–T_ increases, the trajectories that undergo seam-crossing back to the triplet result from a partially relaxed adsorbate, which leads to reduced translational energy in the outgoing atom. When *τ*_S–T_ = 100 fs, the simulated kinetic energy distribution agrees well with experiment. We caution against taking this value of *τ*_S–T_ as an accurate estimate of the actual singlet–triplet crossing delay. It represents the time required for the nascently adsorbed O atom to lose sufficient kinetic energy to undergo a spin-flip transition and, due to the small size of our model system, the energy relaxation rate is probably not quantitatively accurate. What is clear is that a prompt spin relaxation, *τ*_S–T_ = 0, is incompatible with the experimental results. Furthermore, the ability of the model to reproduce the experiment only with a substantial singlet–triplet delay suggests that the spin–orbit coupling is small, consistent with the small atomic numbers of the constituting atoms. Further discussion of the validity of the ad hoc model, including possible failures, is provided in Supplementary Sections 2.5 and 2.6.

## Conclusions

Using a novel high-resolution state-to-state scattering approach, we have experimentally investigated the spin-state-selective scattering of O atoms and their adsorption to HOPG to explore the spin-dependent reactivity and spin-flipping dynamics in surface chemistry. Detailed DFT and MD calculations on high-dimensional PESs machine-learned based on DFT data have been performed to gain a more in-depth understanding of the experimental observations. Both experiment and theory reveal large sticking probabilities for O(^1^D), which are attributed to the formation of a surface-bound epoxide, with ~2-eV binding energy to the surface relative to the triplet asymptote. The nascent epoxide adsorbate formed by collision of O(^1^D) at the surface possesses ~3.3 eV of vibrational energy (as obtained from the singlet binding energy relative to the O(^1^D) asymptote in Fig. 1) and rapidly relaxes below the desorption asymptote. The only mechanism by which O(^1^D) may return to the gas phase involves a spin flip to the triplet state, which is indeed observed in our experiment. Semi-empirical dynamics simulations show that this spin flip must occur after the impinging oxygen atom dissipates a substantial portion of its energy to the HOPG surface, but before it reaches thermal equilibrium.

Sticking probabilities for O(^3^P) are smaller than those of O(^1^D) but are still appreciable under the conditions explored in this work. The fact that the experimental angular and kinetic energy distributions are consistent with the theoretical simulations performed using the triplet PES allows us to conclude that triplet → singlet → triplet conversion does not readily occur at the surface during direct scattering trajectories at incidence energies around 0.34 eV. The most likely sticking mechanism involves dissipation of incidence kinetic energy on the triplet PES, followed by spin relaxation to the lower singlet state on longer timescales.

These conclusions are consistent with the scattering results of atomic oxygen from the 94% O(^1^D) CO_2_ photolysis source, where the scattering kinetic energy distribution of the O(^1^D) → O(^3^P) channel involves hyperthermal yet still relatively low translational energies in comparison to the nearly 2 eV of incidence electronic energy (Fig. 3 and Supplementary Fig. 6). Because the seam of intersection is near the triplet asymptote, the observed energy of the scattered O(^3^P) closely reflects its kinetic energy at the seam of intersection. The high sticking coefficients (Fig. 4) are consistent with this picture. The inefficiency of spin relaxation for incident O(^3^P) atoms limits their likelihood of sticking to the surface, whereas the inability of O(^1^D) to convert to O(^3^P) when the kinetic energy at the seam of intersection is too high leads to enhanced O(^1^D) sticking. These observations underscore the two important factors in spin-flipping dynamics in surface chemistry, namely the efficiency of vibrational energy dissipation and the strength of the spin–orbit coupling. The picture that emerges from this study is that spin-forbidden transitions do occur in this system, but with low probabilities.

## Methods

**Extended Data Fig. 1:**
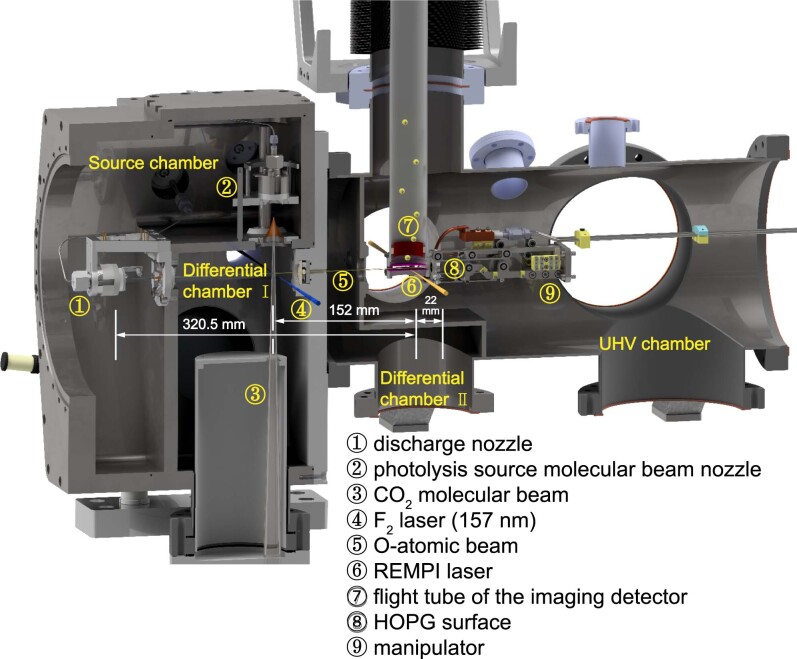
Schematic of the experimental setup of the atomic beam scattering apparatus. The flight distance from the discharge source (1) to the REMPI probe laser (6) is 320.5 mm, whereas the distance from the photolysis region (overlap of the CO_2_ molecular beam (3) with the focused F_2_ laser (4)) to the REMPI laser is 152 mm. The incident atomic beam flies from left to right across two differentially pumped chambers before entering the UHV chamber, where it impinges upon an HOPG surface (8) mounted on a manipulator (9). The surface is located 22 mm from the REMPI laser. Oxygen ions are extracted into a flight tube (7) through which they are sent upward towards an imaging detector (not shown). See Section 1.1 of the Supplementary Information for further details.

The experimental set-up is shown in Extended Data Fig. 1. A photolysis source was used to generate an incident O-atom beam with a high O(^1^D) fraction, and an electric discharge source was used to generate an incident beam with a high O(^3^P) fraction.

### O-atom photolysis source

To generate well-defined O-atom beams comprising predominately O(^1^D), we used 157-nm radiation from an F_2_ excimer laser for photolysis of CO_2_. The 157-nm photolysis of CO_2_ occurs via two channels^30^:

$$\begin{array}{*{20}{c}} {{{\mathrm{CO}}}}_2\left( {\,}^1{{{\mathrm{{\Sigma}}}}}_{{{\mathrm{g}}}}^{+ } \right) + hv \to {{{\mathrm{O}}}}\left({\,}^1{{{\mathrm{D}}}} \right) + {{{\mathrm{CO}}}}\left( {\,}^1{{{\Sigma}}}^{+} \right) & {\left( {{{{\mathrm{channel}}}}\,{{{\mathrm{I}}}},\,94\% } \right)} \end{array}$$

$$\begin{array}{*{20}{c}} {{{{\mathrm{CO}}}}_2\left( {\,}^{1}{{{{{\Sigma}}}}}_{{{\mathrm{g}}}}^{+} \right) + hv \to {{{\mathrm{O}}}}\left( {\,}^3{{{\mathrm{P}}}} \right) + {{{\mathrm{CO}}}}\left( {\,}^{{1}}{{{\mathrm{{\Sigma}}}}}^{+} \right)} & {\left( {{{{\mathrm{channel}}}}\,{{{\mathrm{II}}}},\,6\% } \right)} \end{array}$$

The measured translational energy distribution of the O(^1^D) component is bimodal, with broad peaks at 0.065 and 0.23 eV, whereas the minor O(^3^P) component exhibits a single peak at ~1.5 eV.

### O-atom discharge source

The discharge source makes use of a home-built pulsed solenoid valve equipped with a homemade pulsed d.c. discharge device^31^ based on the design by Lu and colleagues^32^. We measured the beam-state composition to be 87 ± 2% O(^3^P) and 13 ± 2% O(^1^D). Both spin states exhibit broad translational energy distributions peaking at 0.36 eV with a full-width at half-maximum (FWHM) of 0.2 eV (Supplementary Sections 1.1 and 2.1 provide more details).

### Scattering experiments

Scattering experiments were performed from a pristine HOPG surface (grade ZYA, mosaic spread of 0.4 ± 0.1°), held at 298 K and mounted downstream of the beam sources in an ultrahigh-vacuum chamber. The incident and scattered O atoms were detected state-selectively by resonance-enhanced multiphoton ionization (REMPI), using a pulsed, tunable UV dye laser. Ions were detected mass-selectively using a home-built imaging detector, which directly provides the velocity vector of each O atom that undergoes in-plane scattering, following the approach of refs. ^33–36^ (see Supplementary Section 1.1 for more details).

### Incoming/outgoing correlation ion imaging

**Extended Data Fig. 2:**
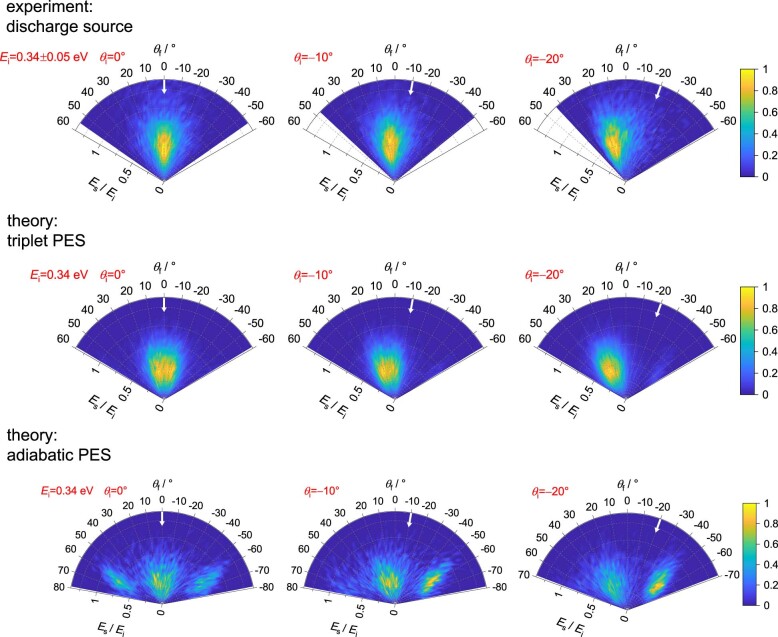
Comparison of Experiment to MD simulations on two PESs. The experimental kinetic energy and angular distribution (top row) obtained for O atoms from the O_2_ discharge source after scattering from HOPG. Scattered atoms were detected in the ^3^P_2_ state. The plotted distribution corresponds to those atoms assigned to an incidence energy range of *E*_i_ = 0.34 ± 0.05 eV via the incoming/outgoing correlation method (Section 1.2 of the Supplementary Information). The results of MD simulations on the triplet PES (middle row) and adiabatic PES (bottom row) are shown for comparison. The simulations were performed at an incidence energy of precisely 0.34 eV. The radial coordinate corresponds to the final kinetic energy divided by the nominal incidence energy (0.34 eV). The left, center, and right columns correspond to incidence angles of 0°, −10°, and −20°, respectively. The incidence angle is indicated by a white arrow for clarity. Source data

The molecular-beam sources of spin-state-enriched O atoms used in this work exhibit broad and irregular velocity distributions, a common problem in molecular beam chemistry that typically prevents high-resolution transitional energy measurements. We have overcome this problem by combining pulsed molecular and laser beams with ion imaging. Controlling the delay time between the pulsed laser and pulsed molecular beam acts as a time-of-flight velocity selection of the incoming atoms. Simultaneously, velocity information about the scattered outgoing atoms is obtained from ion imaging. Combining this incoming/outgoing velocity information allows us to determine the incidence velocity of the O atom connected with individual scattered atoms detected in the ion image. Specifically, for every ion detection event seen in the ion image, we simultaneously record the time *t*_probe_ at which the REMPI laser fired relative to the firing of the atomic beam source. The position in the ion image tells us the velocity vector of the outgoing O atom **v**_f_ and, together with the distance from the REMPI detection point to the surface, *d*_REMPI–surface_, we can calculate the time at which the detected O atom departed the graphite surface:

1 $${t_{{{{\mathrm{depart}}}}} = t_{{{{\mathrm{probe}}}}} - d_{{{{\mathrm{REMPI}}}} - {{{\mathrm{surface}}}}}/v_{z,\,{{{\mathrm{f}}}}}}$$

where *v*_*z*__, f_ is the component of **v**_f_ along the incident atomic beam axis. Assuming negligible residence time, the arrival time of the atom at the surface is *t*_arrive_ = *t*_depart_. This assumption is consistent with the direct scattering mechanisms observed in our work, which exhibit memory effects of the incidence angle and kinetic energy (Figs. 2 and 3 and Extended Data Fig. 2). With the measured distance from the beam source to the surface, *d*_source–surface_, we obtain the incident atom’s speed as

2 $${v}_{{{\mathrm{i}}}} = {d}_{{{{\mathrm{source}}}} - {{{\mathrm{surface}}}}}/t_{{{{\mathrm{arrive}}}}}$$

In this way, we can determine the incident O-atom speed for each scattered atom seen in the ion image. By recording ion images at all values of *t*_probe_ where ions can be detected in the image, we map out distributions of **v**_f_ at particular values of *v*_i_ within the broad distribution of the incident beam. This multiplexing concept allows us to use ‘bad’ O-atom sources with broad speed distributions, but well-defined spin ratios to control incidence electronic excitation. (Supplementary Section 1.2 provides more details.)

### DFT

DFT calculations of atomic oxygen on graphene were performed using the Vienna Ab initio Simulation Package^37,38^. The Perdew–Burke–Ernzerhof functional was used to calculate the exchange-correlation energy^39^. The HOPG surface was approximated by a single graphene layer with a unit cell of 32 carbon atoms, which should be sufficient for low-energy collisions. Unlike the conventional spin-relaxed treatment where the lowest-energy spin state is followed, we constrained the spin of the system by fixing the number of unpaired electrons. These spin-constrained DFT data permit the construction of spin-specific PESs for surface systems. These PESs with 99 dimensions were fit using the Embedded Atom Neural Network method^40^. The triplet and singlet PESs were then used to reconstruct the adiabatic PES by taking the lower energy of the two PESs. These PESs were used as the basis for spin-conserving and spin-changing MD simulations of atomic scattering. Supplementary Section 1.3 provides more details.

## Online content

Any methods, additional references, Nature Portfolio reporting summaries, source data, extended data, supplementary information, acknowledgements, peer review information; details of author contributions and competing interests; and statements of data and code availability are available at 10.1038/s41557-023-01204-2.

## Supplementary information

Supplementary Information Supplementary Figs. 1–11, Methods, Results and References.

## Data Availability

A repository containing data plotted in the supplementary figures, as well as atomic coordinates for the configurations shown in Fig. 1, is available at 10.5281/zenodo.7743197 (ref. ^41^). Source data are provided with this paper.

## Source data

Source Data Fig. 1 Statistical source data.
Source Data Fig. 2 Statistical source data.
Source Data Fig. 3 Statistical source data.
Source Data Fig. 4 Statistical source data.
Source Data Fig. 5 Statistical source data.
Source Data Extended Data Fig. 2 Statistical source data.

## Code availability

The Embedded Atom Neural Network code is available at https://github.com/zhangylch/REANN, and the VENUS code at https://www.depts.ttu.edu/chemistry/Venus/index.php. A tarball (O-HOPG_Theory_Files)—containing the Vienna Ab initio Simulation Package input files, the Venus input file, some representative trajectories, as well as a readme file explaining the data—can be downloaded at 10.5281/zenodo.7743197 (ref. ^41^).
