# Supplementary material for: Spin-dependent reactivity and spin-flipping dynamics in oxygen atom scattering from graphite
Source: Nat Chem. 2023 May 22;15(7):1006–11. doi: 10.1038/s41557-023-01204-2 (PMC10322699; doi:10.1038/s41557-023-01204-2)
Supplement: Supplementary file 1 — Supplementary Figs. 1–11, Methods, Results and References. [file 41557_2023_1204_MOESM1_ESM.pdf]

nature chemistry

Article https://doi.org/10.1038/s41557-023-01204-2

Spin-dependent reactivity and spin-flipping dynamics in oxygen atom scattering from graphite

In the format provided by the authors and unedited

Contents

1 **Methods** 3

1.1 *Experimental* 3

1.2 *Incoming/Outgoing Correlation Ion-imaging: high resolution scattering with "bad beams"* 5

1.3 *Computational methods* 9

2 **Results** 9

2.1 *Scattering experiments with the O_2 discharge source.* 9

2.2 *O(^{1}D) Scattering experiments with the CO_2 photolysis source.* 11

2.3 *Theoretical results for O(^{3}P) scattering.* 12

2.4 *Experimentally determined sticking probabilities.* 14

2.5 *Theoretically determined sticking probabilities.* 17

2.6 *Theoretical simulations of O(^{1}D)→O(^{3}P) spin-flipping dynamics.* 18

3. **References** 20

List of Supplementary Figures

1. Expanded schematic drawing of the O-atom discharge source.
2. Arrival-time dependence of the incidence kinetic energy in scattering experiments performed with the photolysis source.
3. An illustration of how velocity information is used to convert the raw arrival time distribution of scattered atoms to a distribution of departure times from the surface, t_{depart}.
4. Arrival-time dependence of the incidence kinetic energy in scattering experiments performed with the discharge source.
5. Scattering characteristics of the O^{3}P→^{3}P channel.
6. Overall kinetic energy distributions of incident and scattered atoms in the O(^{1}D)→O(^{3}P) channel
7. Kinetic energy distributions of incident and scattered atoms from Channel II of the CO_2 photolysis beam undergoing O(^{3}P)→O(^{3}P) scattering.

8. Turning point distribution of in-plane scattering trajectories on the adiabatic PES for E_i = 0.34 eV.

9. Two-dimensional cuts of the adiabatic PES and triplet PES.

10. In-plane spatial distributions of incident and scattered O atoms.

11. Molecular Dynamics including spin flipping.

1 Methods

1.1 Experimental

Extended Data Fig. 1 shows an overview of the experimental setup. The discharge nozzle is directed along the atomic beam axis with the discharge region 342.5 mm from the HOPG surface. The molecular beam nozzle for photolysis is mounted at 90° to the beam axis with the photolysis region 174 mm from the surface. The REMPI probe laser is focused 22 mm in front of the surface.

Discharge source. Supplementary Fig. 1 shows a schematic of the components of the discharge source. In the discharge region there is a conical electrode and a ground electrode. A ceramic electrode holder and a ceramic electrode spacer are installed between the two electrodes. A −1200 V pulse with 20 µs width is applied to a copper ring attached to the conical electrode, shown in yellow color. An expansion of O_2 seeded in helium passes through the discharge region, where atomic oxygen is formed. The discharge current (~40 mA) is monitored during the discharge. During the discharge a simmer voltage of −1500 V is applied to a copper wire brush mounted off-axis 2.5 cm downstream of the orifice. Electrons that are emitted from the brush stabilize the pulsed discharge current by pre-ionization (<100 ns temporal jitter and 1% amplitude fluctuation).

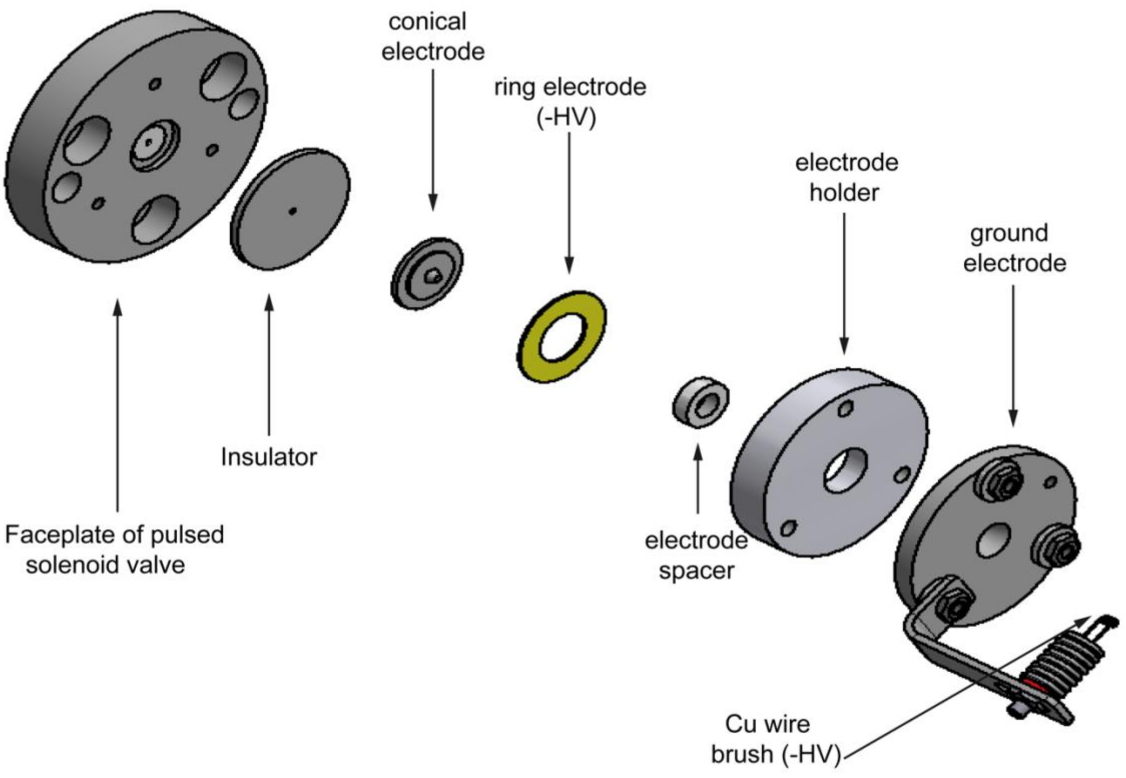

Supplementary Fig. 1: Expanded schematic drawing of the O-atom discharge source.

Photolysis source. For the photolysis process a molecular beam of CO_2 is introduced to the source chamber by a home-built pulsed solenoid valve based on the Even-Lavie design.[1] This molecular beam is collimated by a 2 mm skimmer and subsequently photodissociated by a 157 nm F_2 laser (Coherent LPF Pro 205 F 400 VAC, about 20 mJ pulse energy), focused by a $f = 400$ mm lens, to produce the atomic oxygen beam, which is perpendicular to the pulsed molecular beam and VUV laser.

UHV Chamber and Detector. The atomic oxygen enters the UHV chamber through a 3 mm × 1 mm (horizontal × vertical) rectangular aperture before impinging on the target surface. Prior to each set of scattering experiments, the surface is annealed by electron bombardment heating at 450 ℃ for 30 minutes. The cleanliness and structure of the surface is characterized by low-energy electron diffraction (LEED) and Auger electron spectroscopy (AES). Under the conditions in which experiments were performed, the LEED pattern and AES spectrum were consistent with pristine sp^2 carbon—the oxygen coverage was below the AES detection threshold.

The resonance-enhanced multiphoton ionization (REMPI) detection of the incident and scattered O-atoms makes use of a tunable UV dye laser (Sirah Cobra Stretch CBST-G-18 + THU-205-N), operated with DCM, Rhodamine B, and Rhodamine 101 dyes and pumped by the second harmonic of a Nd:YAG laser (Innolas Spitlight 600 S/N P1754) operated at a repetition rate of 50 Hz. The fundamental is frequency tripled by a pair of BBO crystals yielding the desired ionizing radiation in the 200 to 240 nm range with a pulse energy of 0.4–2.0 mJ and a pulse duration of 8 ns. The laser beam is focused by a 250 mm lens into the region between the repeller and extractor of the detector. The focusing lens is mounted on a motorized translation stage so that the focus can be scanned along the laser propagation direction. During scattering experiments, the laser focus is continuously scanned back and forth so that the effective detection efficiency is uniform along the laser propagation direction. The *J*-resolved detection of $\mathrm{O}(^3\mathrm{P}_{2,1,0})$ is achieved by (2+1) REMPI via the $3\mathrm{p}\ ^3\mathrm{P}$ intermediate state at wavelengths of 225.654, 226.059 and 226.233 nm, respectively [2], and $\mathrm{O}(^1\mathrm{D})$ atom is detected by (2+1) REMPI via the $^1\mathrm{F} \leftarrow {}^1\mathrm{D}$ transition at 203.812 nm.[3]

The laser ionization takes place between a pair of 45 mm diameter extractor and repeller grids that are separated by 5 mm. After ionization, an extraction delay of typically 0.1–2 μs is introduced before pulsing the repeller grid to +2500 V. The extraction delay results in slice imaging,[4,5] which selects only the component of the atoms that scatter in the plane defined by the incident atomic beam and probe laser axes. Mass selection is achieved by pulsing the MCP voltage, and the two-dimensional velocity is extracted from the ion image, obtained either in

spatial imaging mode (without Einzel lens) or velocity mapping mode (with the Einzel lens turned on). The time-of-flight tube to the detector has a length of 44 cm. The scattering angular distribution that can be imaged at a given time is limited by the detector cutoff to approximately $\pm 25°$ from the surface normal. However, the detector can be translated along the probe laser propagation direction to increase the measurable range to $-45°$ to $+65°$ relative to the surface normal.

1.2 Incoming/Outgoing Correlation Ion-imaging: high resolution scattering with "bad beams"

Most previous studies using energy-resolved atomic beam scattering experiments have relied on diatomic molecules as photolysis precursors because energy and momentum conservation lead to sharp incidence kinetic energy (E_i) distributions. Our CO_2 photolysis and O_2 discharge sources lead to incident O-atom beams with broad E_i distributions, but velocity-resolved detection allows us to probe significantly below the energy resolution afforded by the incident beam. In particular, since the photolysis occurs in a narrow time window (20 ns) and well-defined spatial region (~2 mm broad), different incidence velocity components spread out during the long flight distance from the photolysis region to the surface (174 mm) and reach the surface at different times. This is illustrated in Supplementary Fig. 2. The center panel of Supplementary Fig. 2 shows the ion image of incident oxygen atoms from the CO_2 photolysis source, probed at the REMPI laser position $d_{\mathrm{REMPI-surface}}$ = 22 mm in front of the surface at three different photolysis-probe laser delays. The arrival time-dependence of the incidence velocity that was obtained from the ion images was found to match the result calculated from the 152 mm flight distance between the photolysis and probe laser divided by the photolysis-probe laser delay (Eq. 2 of the main article). Since the incidence velocity (v_i) of each atom is known, it is trivial to calculate its arrival time at the surface from

$$t_{\mathrm{arrive}} = t_{\mathrm{probe}} + \frac{d_{\mathrm{REMPI-surface}}}{v_i} \quad (1)$$

which allows us to construct a plot of $\langle E_i \rangle$ vs t_{arrive} (Supplementary Fig. 2, left-hand side).

VMI images of the scattered atoms are obtained as a function of the photolysis-probe laser delay, t_{probe}. The total flux of scattered atoms detected at each delay is shown in the left-hand panel of Supplementary Fig. 3, and representative ion images obtained at three delays are shown at the top of Supplementary Fig. 3. Since the velocity components of each scattered atom are also measured at each probe laser delay, the time at which each REMPI detected atom left the surface is calculated from

$$t_{\text{depart}} = t_{\text{probe}} - \frac{d_{\text{REMPI-surface}}}{v_{z,f}} \tag{2}$$

where $v_{z,f}$ is the component of velocity of the scattered atom along the incident beam direction. The boxed regions shown in the ion images of Supplementary Fig. 3 represent the assignment of ions into 10 μs histogram intervals of t_{depart}. The overall t_{depart} distribution (right-hand panel of Supplementary Fig. 3) is obtained by summing the histograms obtained at each value of t_{probe}.

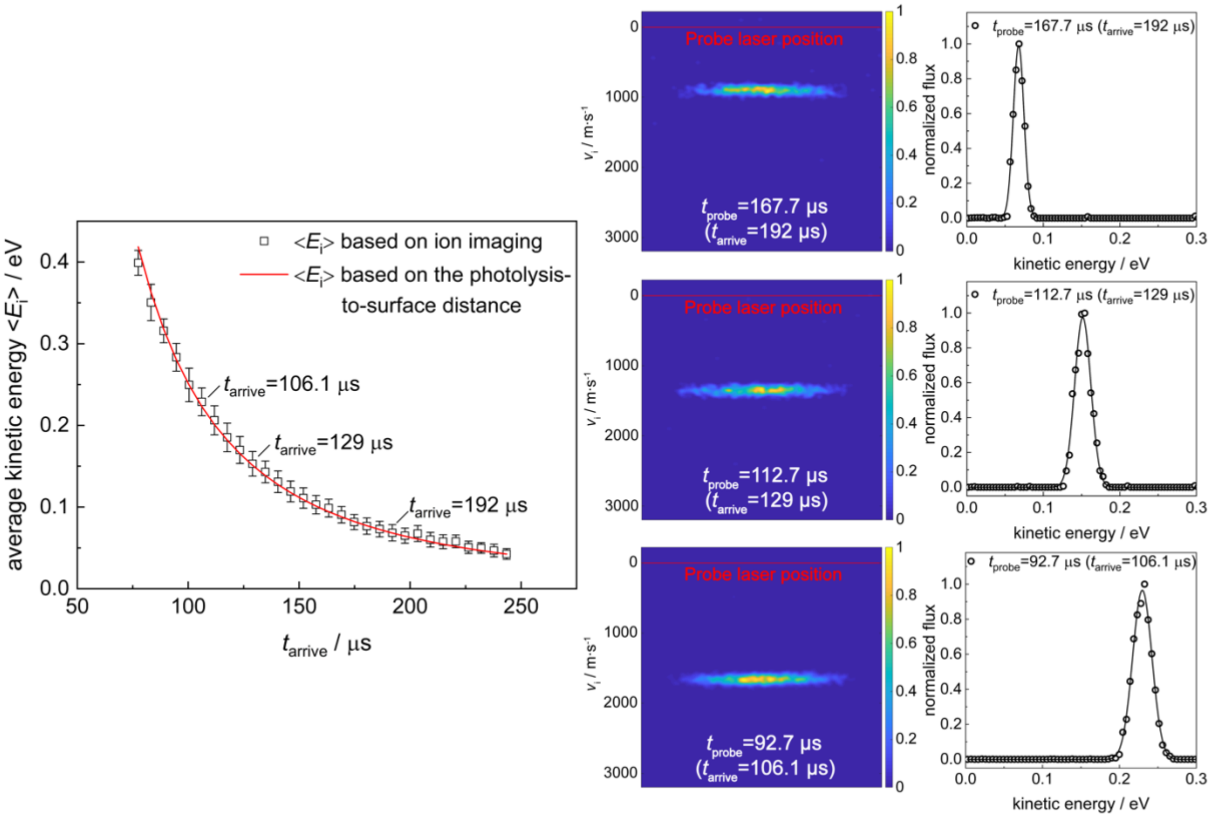

Supplementary Fig. 2: Arrival-time dependence of the incidence kinetic energy in scattering experiments performed with the photolysis source. The relationship between arrival time at the surface and the expectation value of incidence kinetic energy is shown in the left panel for the O atom beam obtained from the CO_2 photolysis source. The black square markers indicate the value of $\langle E_i \rangle$ obtained from ion imaging and the error bars indicate the FWHM of the instantaneous E_i distribution obtained at each delay. The red curve shows the expected arrival time dependence of kinetic energy derived from the experimental geometry. The ion images at $t_{\text{probe}} = 92.7\ \mu s$, $t_{\text{probe}} = 113\ \mu s$, and $t_{\text{probe}} = 168\ \mu s$ are illustrated in the middle panel, and their corresponding kinetic energy distributions are shown in the right panel.

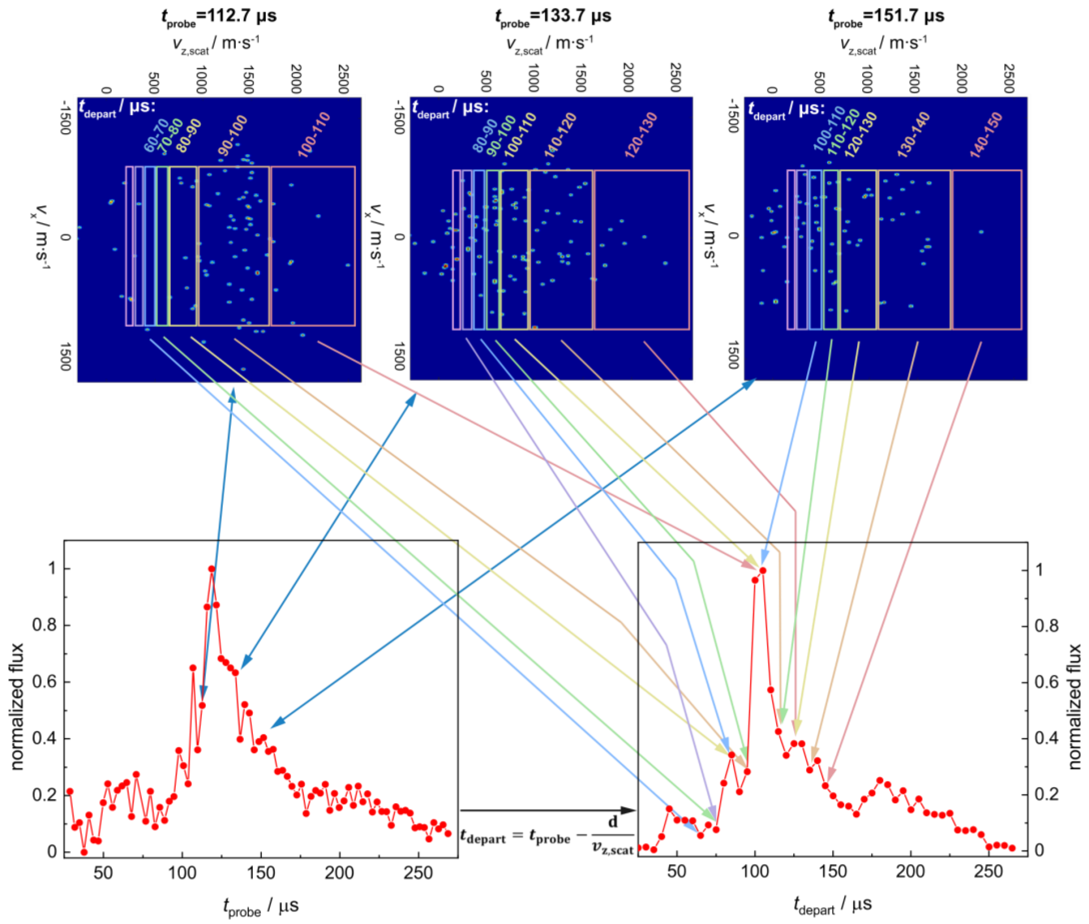

Supplementary Fig. 3: An illustration of how velocity information is used to convert the raw arrival time distribution of scattered atoms to a distribution of departure times from the surface, t_{depart}. The lower left panel shows the total flux of scattered O ($^3P_2$) atoms obtained using the CO_2 photolysis source as a function of the photolysis-probe laser delay, t_{probe}. VMI images obtained at three representative delays are shown at the top. For each ion in the image, the departure time from the surface, t_{depart}, is calculated from $v_{z,f}$ using Eq. 2. The rectangular boxes drawn over the ion images represent 10 μs histogram intervals for t_{depart} (as labeled). The overall t_{depart} distribution obtained by summing the histograms for every image is shown in the lower right panel. The contribution of ions to different t_{depart} intervals is indicated by colored arrows. Due to the broad distribution of scattered velocities, ions from images obtained at multiple different t_{probe} delays contribute to each interval of t_{depart}.

For direct scattering phenomena described in the current work, the sub-picosecond residence time at the surface can be considered negligible, which allows us to equate t_{arrive} with t_{depart} and assign a value of $\langle E_i \rangle$ to each scattered atom based on the arrival-time dependence of $\langle E_i \rangle$, which can be read from the left panel of Supplementary Fig. 2. A much higher energy resolution than the width of the E_i distribution is obtained by selecting only those scattered atoms assigned to a particular $\langle E_i \rangle$ interval.

The approach can also be applied to experiments involving the discharge source with the only difference being that the arrival times of different incidence kinetic energy components are less well resolved (~20% E_i resolution), since the O atoms are generated over a broader time window (~20 μs) and in a broader spatial region (~4 mm) compared to the photolysis source. Supplementary Fig. 4 illustrates the relationship between arrival time at the surface, t_{arrive}, and incidence kinetic energy, E_i, obtained with the discharge source. The central panel

shows ion images of the incident atomic beam acquired at three different t_{probe} delays, defined as the delay between the maximum of the discharge current pulse and the firing of the REMPI probe laser. For each image, the expectation value of t_{arrive} is calculated from Eq. 1 using the measured velocity distribution. The distance $d_{REMPI\text{-}surface}$ between the probe laser and surface was 22 mm. The incidence kinetic energy distribution corresponding to each image is shown in the right-hand side of Supplementary Fig. 4, and the relationship between t_{arrive} and E_i is shown in the left-hand panel. Vertical error bars represent the FWHM of the instantaneous distribution of E_i of atoms impinging on the surface at each point in time. There is a clear time dependence of $\langle E_i \rangle$, but the energy resolution is only approximately 20%, due primarily to the finite ($\sim 20\ \mu$s) length of the discharge current pulse. The red curve through the data represents the expected value of $\langle E_i \rangle$, calculated from the 342.5 mm distance between the discharge source and the surface and the time delay (Eq. 2 of the main text).

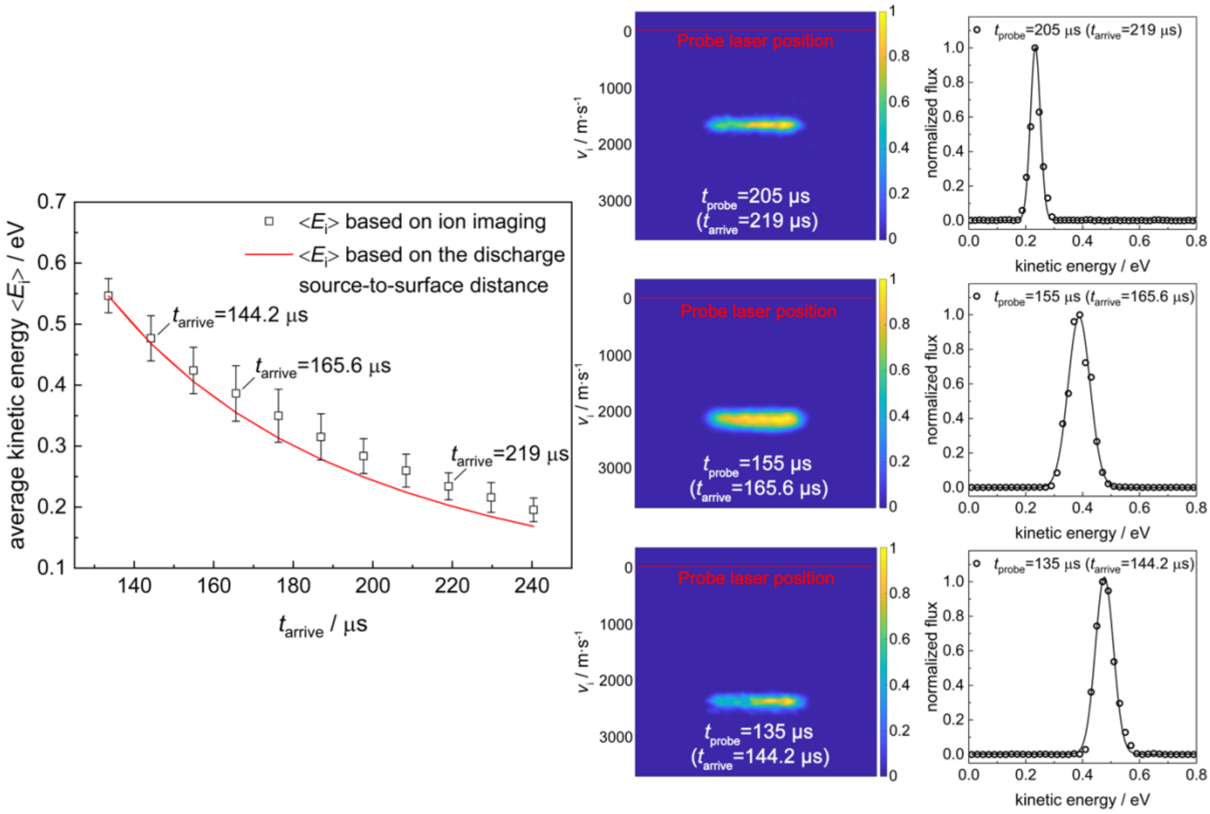

Supplementary Fig. 4: Arrival-time dependence of the incidence kinetic energy in scattering experiments performed with the discharge source. The center panel shows ion images of incident O $^3P_2$ atoms from the discharge source obtained at different t_{probe} delays. The corresponding kinetic energy distributions are displayed in the right-hand side of the figure and the expectation value of arrival time at the surface for each group of atoms is indicated in the figure. The left-hand panel shows the expectation value of incidence kinetic energy as a function of arrival time at the surface. Vertical error bars represent the FWHM of the instantaneous distribution of incidence energies derived from the measurement. The red curve shows the expected time dependence of E_i, based on the distance from the discharge source to the surface.

1.3 Computational methods

Density Functional Theory (DFT). The computational results were obtained from plane-wave DFT calculations performed using the Vienna Ab initio Simulation Package (VASP).[6,7] The PBE functional is used to calculate the exchange-correlation term based on the generalized gradient approximation (GGA).[8] The electron−ion interaction is described by the projector augmented-wave method (PAW).[9] The energy cutoff of the plane-wave basis is set to 400 eV. The HOPG surface is modeled by periodic slabs with one-layer 4×4 cell containing 32 movable C atoms and a vacuum space of 15 Å in the *z*-direction. The Brillouin zone is sampled with a 2 × 2 × 1 *k*-point grid.

The spin-constrained triplet calculations are carried out using spin-polarization with NUPDOWN=2, while the singlet calculations are performed with spin-unpolarized DFT.

The O atom adsorption energy is calculated according to

$$E_{\mathrm{ad}}(\mathrm{O}) = E_{\mathrm{O@Gra}} - E_{\mathrm{O}} - E_{\mathrm{Gra}} \tag{3}$$

where $E_{\mathrm{O@Gra}}$, E_{O} and E_{Gra} are the energy of the whole system, the O atom in gas phase and the isolated graphene sheet.

Ab Initio Molecular Dynamics (AIMD). About 400 AIMD trajectories are calculated with an O atom incidence energy of 1.0 eV along the surface normal at a surface temperature of 300 K. The system is constrained to either the singlet or triplet state throughout the simulation, using the spin-constraining recipe described above. The initial position of the O atom is evenly sampled over the unit cell at 6 Å above the surface.

NN PES fitting. PESs with 33 movable atoms (99 dimensions) are fitted with the Embedded Atom Neural Network (EANN) method,[10] which enforces full permutation symmetry. 120000 points (energies and gradients) are used in the fitting. These points are culled from the AIMD trajectories using a criterium based on the Euclidean distance larger than 0.3 Å. The fitting quality of the singlet and triplet PESs is given by root mean square errors (RMSEs) of 19 and 26 meV, respectively. These two PESs intersect, as shown in Figure 1, and the lower energy one of the two PESs forms the adiabatic spin-relaxed PES.

2 Results

2.1 Scattering experiments with the O_2 discharge source.

Under the conditions used in this experiment, the O-atom discharge source gave rise to an incident O atom beam with a state distribution of ^{3}P:^{1}D=0.867±0.018:0.133±0.018 and ^{3}P$_2$:^{3}P$_1$:

$^3P_0$=0.933±0.038:0.053±0.031:0.014±0.007. The incident beam had a broad kinetic energy distribution peaked at 0.36 eV with a FWHM of 0.2 eV (see Supplementary Fig. 5, left panel). The overall angular and kinetic energy distribution of incident and scattered O $^3P_2$ atoms obtained in the discharge experiment is shown in Supplementary Fig. 5 for three different incidence angles. Using the incoming/outgoing correlation method, we assigned a value of $<E_i>$ to each scattered atom. The solid red curve in Fig. 3d of the main text includes only those atoms with $<E_i>$ in the range 0.23 ± 0.05 eV. Figure 2d of the main text shows the dependence of the mean scattered kinetic energy on the incidence energy.

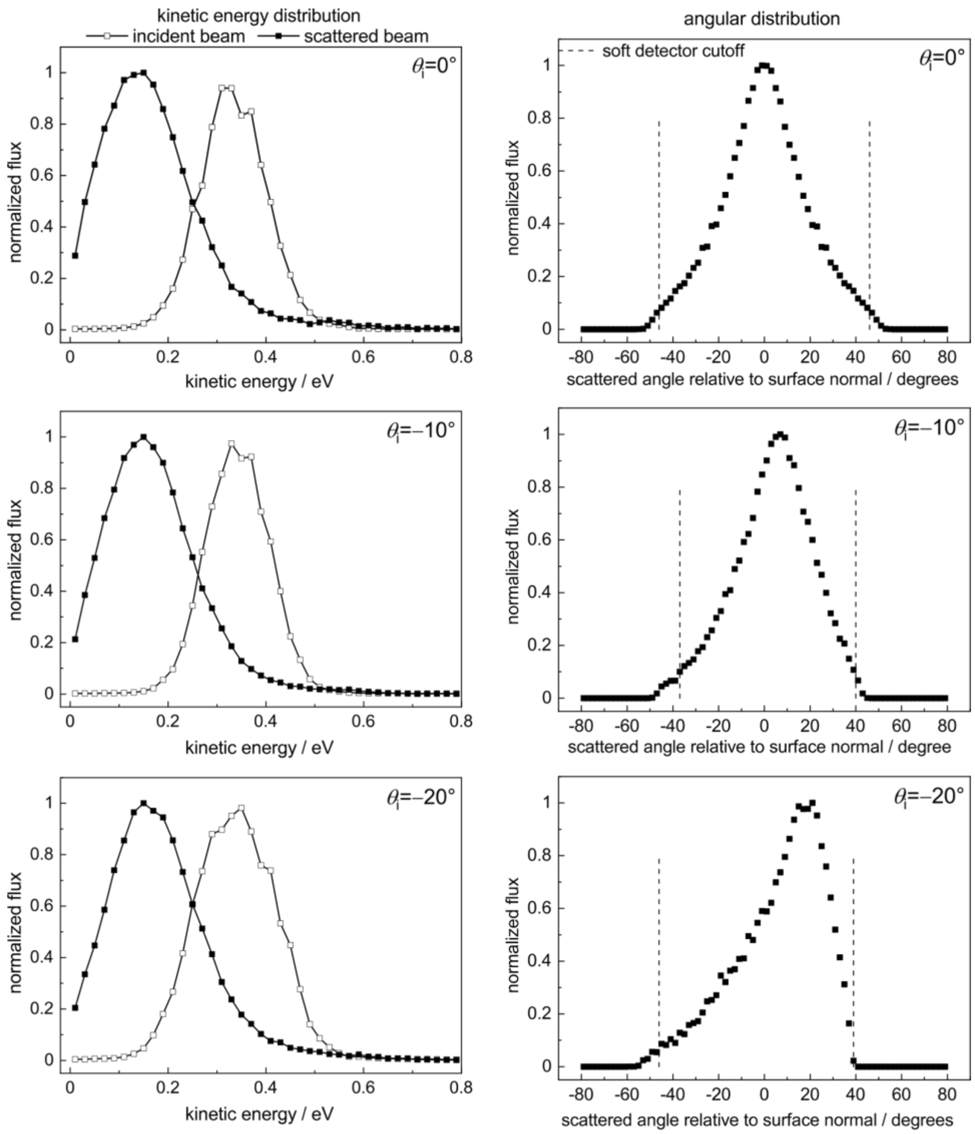

Supplementary Fig. 5: Scattering characteristics of the O^{3}P → ^{3}P channel. Left panel: The kinetic energy distributions of incident O $^3P_2$ atoms generated by running 1% O_2/He at a stagnation pressure of 10 bar through a pulsed discharge source, operated with a discharge voltage of −1200 V and a discharge current pulse width of 20 μs, is shown with open squares and the kinetic energy distribution of O($^3P_2$) atoms scattered from HOPG at normal incidence angle is shown with solid squares. The angular distribution of scattered O($^3P_2$) is shown in the right panel. The dashed vertical lines indicate the angles at which a portion of the ions start to be cut off due to the detector geometry. The portion of the angular distribution between the dashed lines can be considered reliable.

2.2 O(^{1}D) Scattering experiments with the CO_2 photolysis source.

The overall kinetic energy distribution of the incident O(^{1}D) and scattered O($^3P_2$) atoms are shown in Supplementary Fig. 6. The incident O(^{1}D) atoms have a broad kinetic energy distribution with peaks at 0.065 and 0.23 eV, which correspond to formation of the CO co-product in the v=1 and v=0 states, respectively. Both peaks are broadened due to the population of many CO rotational states. The scattered kinetic energy distribution is broad, with a peak at around 0.15 eV. Such a hyperthermal distribution is consistent with a direct scattering mechanism, which occurs on a short timescale. Note that the distribution extends somewhat higher in energy than that of the incident beam, suggesting that some of the incident electronic energy has been converted to kinetic energy. The main text explores this suggestion further.

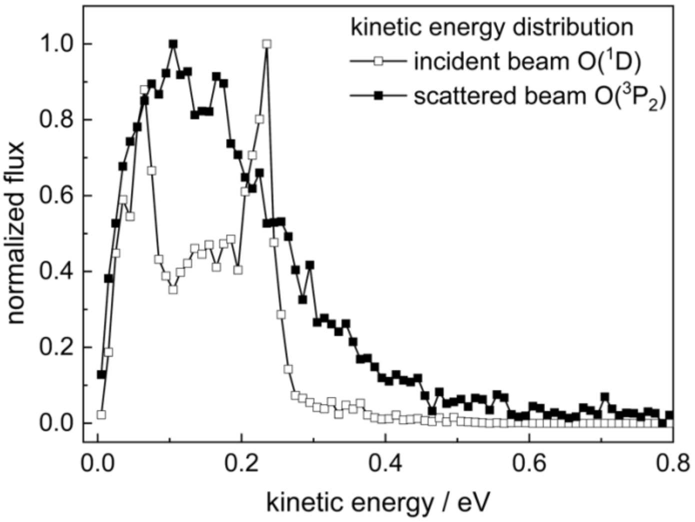

Supplementary Fig. 6: Overall kinetic energy distributions of incident and scattered atoms in the O(^{1}D)→O(^{3}P) channel. The kinetic energy distribution of incident O(^{1}D) atoms obtained from the 157 nm photolysis of CO_2 is shown as open markers. The resulting kinetic energy distribution of O($^3P_2$) state atoms scattered from HOPG at normal incidence angle is shown with filled markers.

For t_{arrive} = t_{depart} times between 30 μs and 90 μs, the contribution of the minor $\text{O}(^3\text{P}) \rightarrow \text{O}(^3\text{P})$ channel is well resolved from the dominant $\text{O}(^1\text{D}) \rightarrow \text{O}(^3\text{P})$ channel (see Fig. 3b of the main text). The kinetic energy distributions of incident and scattered O($^3P_2$) atoms that were assigned to this range of t_{arrive} or t_{depart}, respectively, are plotted in Supplementary Fig. 7. The average incidence kinetic energy $\langle E_\text{i} \rangle = 1.28$ eV and the average scattering kinetic energy $\langle E_\text{s} \rangle = 0.525$ eV. The incidence energy distribution has a width of ~1 eV. We did not attempt to assign scattered atoms to more precise E_i intervals because the signal level in this scattering channel was low, due to the small amount of $\text{O}(^3\text{P})$ produced by this beam source.

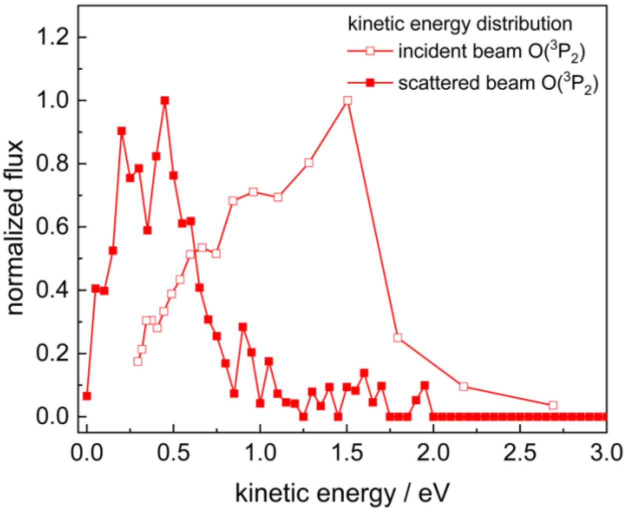

Supplementary Fig. 7: Kinetic energy distributions of incident and scattered atoms from Channel II of the CO_2 photolysis beam undergoing $O(^3P) \rightarrow O(^3P)$ scattering. Open squares show the kinetic energy distribution of incident $O(^3P_2)$ atoms, and filled squares show the kinetic energy distribution of scattered $O(^3P_2)$ atoms that were assigned to $t_{\text{arrive}} = t_{\text{depart}}$ times between 30 μs and 90 μs (corresponding to the early peak in Fig. 3b of the main text).

2.3 Theoretical results for $O(^3P)$ scattering.

MD trajectories for incident O atoms in the ^3P ground state are calculated using two different PESs: the triplet and adiabatic PESs. For all trajectories, the initial kinetic energy is set at 0.34 eV and three initial polar incidence angles ($\theta_i = 0°$, $-10°$, and $-20°$) are chosen in order to compare with experimental results, while the initial azimuthal angle is generated randomly ranging from 0 to 360 degrees. The surface temperature is set at 300 K. Because in the experiment, only in-plane scattering is observed, we also choose in-plane trajectories to make meaningful comparisons. Considering that strictly in-plane trajectories represent only a small fraction of the total trajectories and a very large number of total trajectories are needed to generate enough in-plane trajectories to get converged statistical results, trajectories within ±20 degrees of acceptance angle are counted as in-plane trajectories.

The angular and kinetic energy distributions obtained from the scattering trajectories on the two PESs are compared with the experimental results in Extended Data Fig. 2. (The $\theta_i = -20°$ data is the same as that shown in Fig. 2(a)–(c) of the main text.) Both PESs lead to similar scattering kinetic energy distributions, but to qualitatively different angular distributions and sticking probabilities (see below). The triplet PES resulted in an angular distribution peaked near the specular angle, whereas the adiabatic PES resulted in a bimodal distribution with a second peak at much larger angles. When $\theta_i \neq 0$, backward scattering (toward the incidence direction) is seen. To gain insight into the origin of the backward scattering, the position of the turning point of the scattered trajectories on the adiabatic PES, which is defined as the point when the normal component of the O atom velocity changes direction, is computed and plotted in Supplementary Fig. 8. It was found that most in-plane specular scattering takes place at the

top site (red crosses in Supplementary Fig. 8) and the larger angle scattering occurs at the hollow site (blue crosses in Supplementary Fig. 8). This can be understood from the two-dimensional cuts of the triplet and adiabatic PESs along the *xz* and *yz* planes with all the other dimensions relaxed, as shown in Supplementary Fig. 9. Comparing with the triplet PES, the adiabatic PES is much more attractive and corrugated. The corresponding PES near the top site is relatively flat, leading largely to specular scattering. On the other hand, the strong corrugation near the hollow site results in backward scattering.

Although the predicted back scattering peak occurs near the detector cutoff, we would still expect to be able to experimentally observe indications of this peak at angles between −20 to $-50°$ when $\theta_\mathrm{i} = -20°$ (upper right panel of Extended Data Fig. 2). The absence of any indication of such a peak in the measured angular distribution leads us to conclude that the experiment is more consistent with the simulations performed on the triplet PES, which suggests that triplet→singlet→triplet conversion *does not* readily occur at the surface during direct scattering trajectories at incidence energies around 0.34 eV.

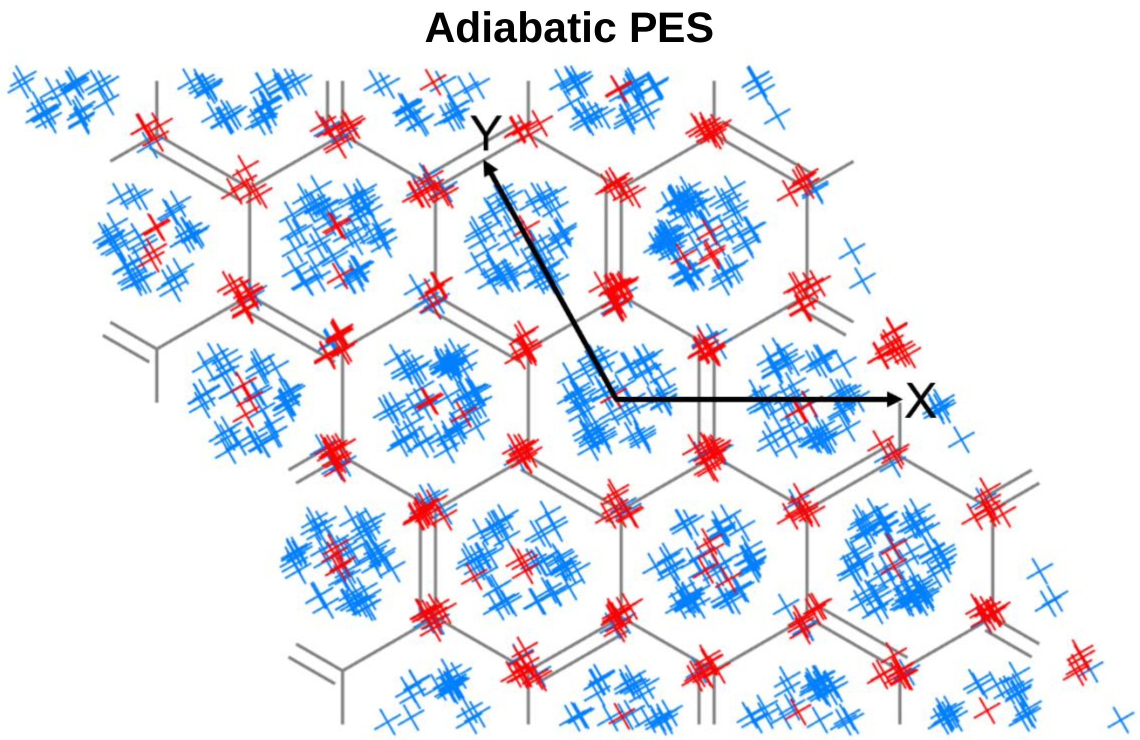

Supplementary Fig. 8: Turning point distribution of in-plane scattering trajectories on the adiabatic PES for $\boldsymbol{E_\mathrm{i}} = \mathbf{0.34}$ eV. The red and blue crosses represent trajectories with near-specular and non-specular scattering angles, which are concentrated at the top and hollow sites, respectively. The *x* and *y* coordinates are defined by the vectors in the surface plane and *z* is the coordinate perpendicular to the surface plane.

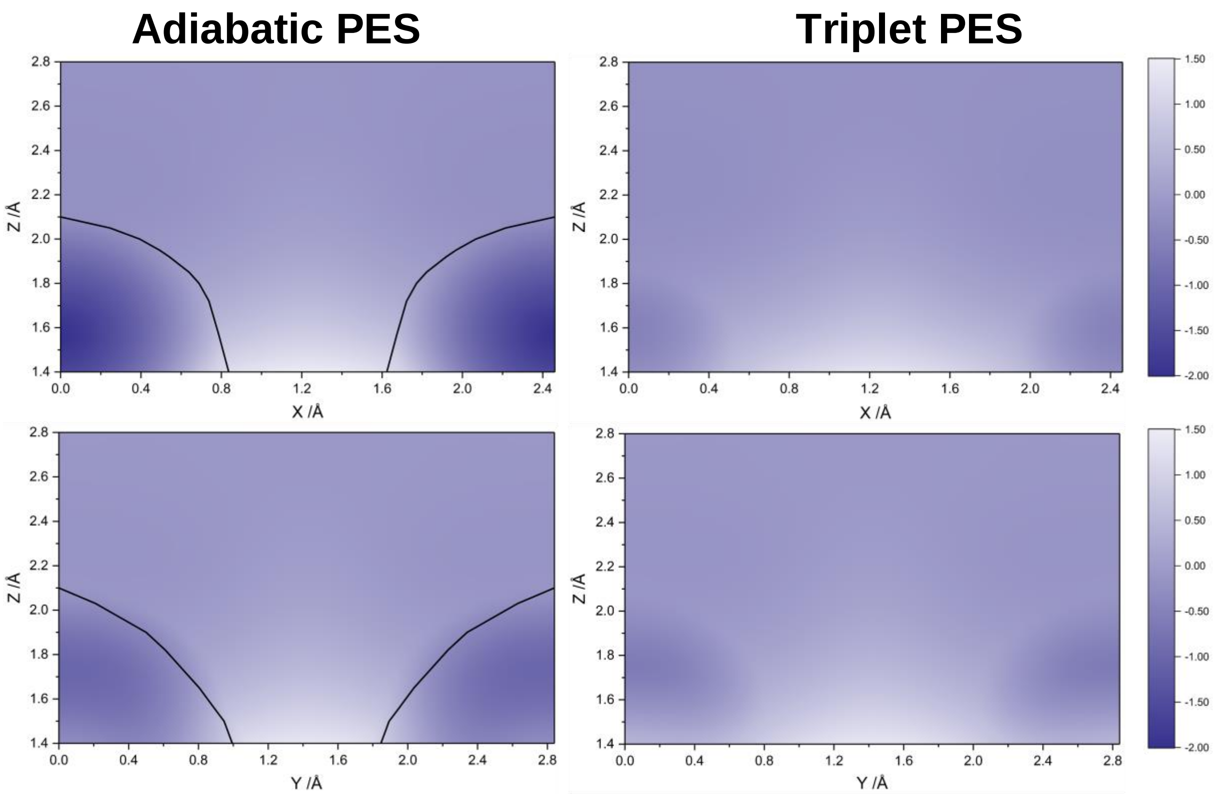

Supplementary Fig. 9: Two-dimensional cuts of the adiabatic PES (left panels) and triplet PES (right panels). The *xz* and *yz* planes are defined as in Supplementary Fig. 8. The triplet-singlet crossing seam is displayed in the left panels as black curves. The energy, in eV, is relative to the triplet asymptote.

2.4 Experimentally determined sticking probabilities.

We obtained the sticking coefficients from experiment by measuring the ratio of the integrated flux of incident and scattered O atoms. Here, we describe the procedure in detail for the CO_2 photolysis source, used to obtain the $O(^1D)$ sticking coefficient. The sticking coefficient for $O(^3P)$ was obtained in a similar manner using the O_2 discharge source. For the CO_2 photolysis source, we measured the incident $O(^3P_2)$ flux and scattered $O(^3P_2)$ flux at the same time, while scanning the probe laser focus along the laser propagation direction. The REMPI scheme, laser power, and nozzle conditions were hence identical for the two measurements. The ratio $O(^1D){:}\,O(^3P_2) = 94\%{:}\,4.2\%$ obtained from the 157 nm photolysis of CO_2 is taken directly from the literature.[11,12] The incident $O(^1D)$ density is equal to the incident $O(^3P_2)$ density multiplied by this ratio.

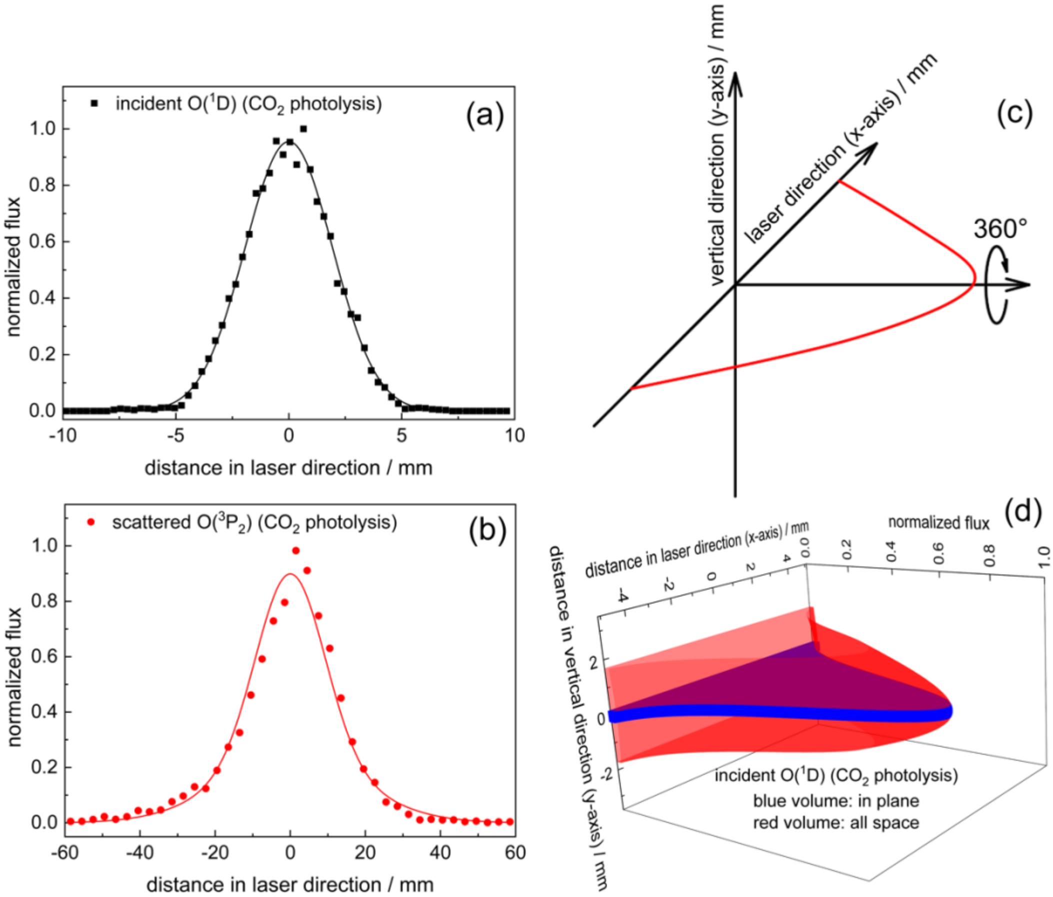

Supplementary Fig. 10: In-plane spatial distributions of incident and scattered O atoms. Panels (a) and (b) show the spatial flux distribution in the laser direction for scattered $O(^3P_2)$ and incident $O(^1D)$ from the CO_2 photolysis source. Panel (c) shows the axis system, where the *x*-axis is laser direction, the *y*-axis corresponds to vertical direction, and the *z*-axis is along the surface normal direction. Panel (d) is the incident O^1D spatial distribution from the CO_2 photolysis source in 3D space.

The experiment measures only the in-plane component of the incident and scattered flux via slice imaging (Supplementary Figs. 10(a) and 10(b)). These distributions were fitted with Gaussian functions, which can be expressed (in arbitrary units) as:

$$f_{\mathrm{i,in\ plane}}(x) = 0.95 \cdot \exp\left(-\frac{x^2}{2\cdot(1.93\ \mathrm{mm})^2}\right) \quad (4)$$

$$f_{\mathrm{scat,in\ plane}}(x) = 0.22 \cdot \exp\left(-\frac{x^2}{2\cdot(18.74\ \mathrm{mm})^2}\right) + 0.68 \cdot \exp\left(-\frac{x^2}{2\cdot(9.07\ \mathrm{mm})^2}\right) \quad (5)$$

The vertical height of the scattered in-plane O-atom signal is determined by the focal waist of the laser. We assume a height of 0.384 mm, which corresponds to an acceptance angle about $\pm1°$ relative to the scattering plane. (Note that the final result is not sensitive to the accuracy of this value due to cancellation of error when taking the ratio between the incident and scattered flux.) The volume of the resulting truncated Gaussian function is calculated from:

$$V = 2\pi a {\sigma_a}^2 \left[\Phi\left(\frac{y_0}{\sigma_a}\right) - \Phi\left(-\frac{y_0}{\sigma_a}\right)\right] \tag{6}$$

where y_0 is the vertical cutoff distance, $\Phi(x) = \frac{1}{\sqrt{2\pi}} \int_{-\infty}^{x} \exp\left(-\frac{t^2}{2}\right) \mathrm{d}t$ is the cumulative distribution function of the standard normal distribution, and σ_a is the Gaussian width parameter.

In order to estimate the scattering probability in three-dimensional space $p_{\mathrm{scat,3D}}$, we assumed that the distribution has cylindrical symmetry about the surface normal and rotated the fitted in-plane distribution over 360° about the surface normal. Such a rotated Gaussian function is given in the coordinate system of Supplementary Fig. 10(c) by:

$$g(x,y) = a \exp\left(-\frac{x^2+y^2}{2{\sigma_a}^2}\right) \tag{7}$$

We used a rectangular orifice ($x \times y$ = 3 mm × 1 mm) in the experiment such that the incident beam is truncated along the vertical direction. We expect the width of the incident atomic beam to be three times wider along the *x*-direction than along the *y*-direction. The incident $\mathrm{O}(^1\mathrm{D})$ spatial distribution of CO_2 photolysis source in 3D space is shown in Supplementary Fig. 10(d). The volume of integration corresponding to the in-plane O atom flux (laser focus size $y = \pm 0.192$ mm) is shaded blue, and the volume of integration corresponding to the overall flux is shaded red. The distance along the laser direction (*x* axis) is about ± 5.1 mm, so the distance along the vertical direction (*y* axis) is about $\pm\frac{5.1}{3}$ mm, and the volume of integration (shaded red), in arbitrary units, is

$$V_{\mathrm{i,3D}} = 2\pi \cdot 0.95 \cdot 1.93^2 \cdot \left[\Phi\left(\frac{\frac{5.1}{3}}{1.93}\right) - \Phi\left(-\frac{\frac{5.1}{3}}{1.93}\right)\right] = 13.82 \tag{8}$$

The laser focus size is ± 0.192 mm, so the volume of integration corresponding to the in-plane flux (shaded blue), in arbitrary units, is

$$V_{\mathrm{i,in\ plane}} = 2\pi \cdot 0.95 \cdot 1.93^2 \cdot \left[\Phi\left(\frac{0.192}{1.93}\right) - \Phi\left(-\frac{0.192}{1.93}\right)\right] = 1.76 \tag{9}$$

The integrated flux in 3D space can be obtained by correcting the in-plane flux component by the ratio between the volume of integration for in plane signal and for 3D signal:

$$\mathrm{flux}_{\mathrm{3D}} = \left(\int f_{\mathrm{i,in\ plane}}(x)\mathrm{d}x\right)\frac{\mathrm{V_{3D}}}{\mathrm{V_{in\ plane}}} \tag{10}$$

The scattering probability is equal to

$$p_{\text{scat,3D}} = \frac{flux_{\text{scat,3D}}}{flux_{\text{i,3D}}} = \frac{\left(\int f_{\text{scat,in plane}}(x)\mathrm{d}x\right)\frac{\mathrm{v}_{\text{scat,3D}}}{\mathrm{v}_{\text{scat,in plane}}}}{\left(\int f_{\text{i,in plane}}(x)\mathrm{d}x\right)\frac{\mathrm{v}_{\text{i, 3D}}}{\mathrm{v}_{\text{i, in plane}}}} \tag{11}$$

The sticking coefficient is calculated from:

$$s_0 = 1 - p_{\text{scat,3D}} \tag{12}$$

In experiments using the discharge source, both the in-plane incident and scattered $\mathrm{O}(^3\mathrm{P})$ fluxes are measured directly. The sticking coefficient for $\mathrm{O}(^3\mathrm{P})$ is then determined from Eqns. 11 and 12 in a manner similar to that described above for $\mathrm{O}(^1\mathrm{D})$. The results are shown in Fig. 4 of the main text.

2.5 Theoretically determined sticking probabilities.

The MD simulations also allow a theoretical determination of the O(^{3}P) sticking probability. On the spin-relaxed adiabatic PES, there was a very high probability of sticking—in about 93% of trajectories, the O atom was still less than 2.0 Angstroms from the surface after 50 ps. Due to the much weaker binding on the triplet surface, the sticking probability was much lower on this surface (about 68% after 50 ps). We note that due to the finite number of atoms in the model graphene, the dissipation of energy is not expected to be complete in the simulation.

The O(^{1}D) simulation with $\tau_{S-T} = 100$ fs yielded a sticking probability of 0.997 at an incidence energy of 0.23 eV. This value is much higher than the experimental value of ~0.7 $\pm$ 0.1. Error might arise due to the uncertainties in electronic structure calculations, in particular the adsorption well depth and the crossing seam between the two states. Such errors are not unexpected with DFT as evidenced by its inability to reproduce the O(^{1}D)-O(^{3}P) energy gap. Another type of error might arise if electronically excited states, which are not considered here, participate in the dynamics due to the coupling of O-atom orbitals to the conduction band of the graphene layer. For example, if the dynamics can take place on electronically excited singlet states, this could shift the location of the intersection with the triplet state, possibly leading to an effective broadening of the seam of intersection. In addition, the *ad hoc* assumption used to treat the spin flipping dynamics and the limited size of the graphene model may also be responsible for the disagreement.

2.6 Theoretical simulations of $O(^1D)\rightarrow O(^3P)$ spin-flipping dynamics.

We performed an MD simulation to consider spin non-conserving $O(^1D)\rightarrow O(^3P)$ trajectories, as described in the main text. Due to intrinsic errors of the DFT calculations, the energy of gas-phase $O(^1D)$ is only about 1.3 eV higher than that of $O(^3P)$. In order to match the experimental energy gap between singlet and triplet O, which is about 2 eV, the singlet PES was shifted upwards by 0.7 eV in our calculations. We used three different values of τ_{S-T} (0, 50 fs, and 100 fs) and performed simulations with 10,000, 50,000, and 700,000 trajectories, respectively. Supplementary Fig. 11 shows the energy difference between the singlet and triplet PESs for points along several scattering trajectories with the spin-flip delay τ_{S-T} set to 100 fs. The inset shows the results for one representative trajectory, plotted alongside the *z*-distance of the O atom above the surface. The O atom reaches the seam of intersection (∼2 Å above the surface) about 200 fs after the start of the simulation. For the next 100 fs, the O atom was constrained to the singlet PES, no matter whether it reaches the intersection or not. After that, at about 400 fs, the O atom jumps to the triplet state once it crosses the seam, leading eventually to desorption from the surface as evidenced by the increasing *z* value. The kinetic energy distributions obtained at different τ_{S-T} delays are described in the main text.

Admittedly, this *ad hoc* model is not expected to quantitatively reproduce the experimental finding. Rather, it is used to test the viability of the nonadiabatic spin-flip mechanism in this system. The results underscore competition of two important timescales, namely the rate of energy dissipation and the rate of nonadiabatic transitions. The former is controlled by the interaction of the impinging oxygen atom with the HOPG, while the latter by the spin-orbit coupling and the velocity of the O atom passing through the seam. The current model is insufficient to provide quantitative information on either rate; more sophisticated theory is needed.

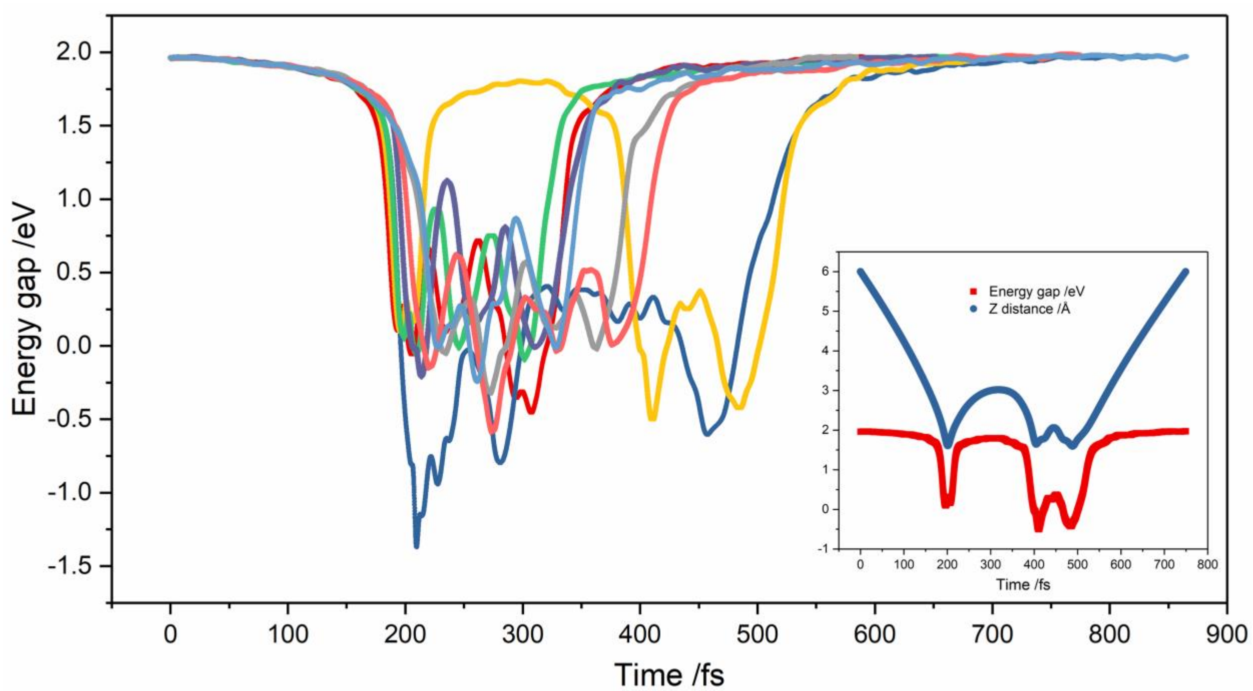

Supplementary Fig. 11: Molecular Dynamics including spin flipping. The energy difference between the singlet and triplet PESs is plotted for several scattering trajectories as a function of time. The O(^{1}D)→O(^{3}P) spin-flip delay τ_{S-T} (see main text) was set to 100 fs. A positive energy gap corresponds to a configuration where the triplet PES is more stable and a negative energy gap corresponds to a configuration where the singlet PES is more stable. The seam of intersection is crossed when the energy gap changes sign. The inset plots the energy gap (red) and z-distance (blue) simultaneously for a representative trajectory.

3. References

1 Even, U., Jortner, J., Noy, D., Lavie, N. & Cossart-Magos, C. Cooling of large molecules below 1 K and He clusters formation. *J. Chem. Phys.* **112**, 8068-8071, doi:10.1063/1.481405 (2000).

2 Bamford, D. J., Jusinski, L. E. & Bischel, W. K. Absolute two-photon absorption and three-photon ionization cross sections for atomic oxygen. *Phys. Rev. A* **34**, 185-198, doi:10.1103/PhysRevA.34.185 (1986).

3 Pratt, S. T., Dehmer, P. M. & Dehmer, J. L. Double-resonance spectroscopy of transitions between autoionizing levels of atomic oxygen. *Phys. Rev. A* **43**, 4702-4711, doi:10.1103/PhysRevA.43.4702 (1991).

4 Gebhardt, C. R., Rakitzis, T. P., Samartzis, P. C., Ladopoulos, V. & Kitsopoulos, T. N. Slice imaging: A new approach to ion imaging and velocity mapping. *Rev. Sci. Instrum.* **72**, 3848-3853, doi:10.1063/1.1403010 (2001).

5 Harding, D. J., Neugebohren, J., Auerbach, D. J., Kitsopoulos, T. N. & Wodtke, A. M. Using Ion Imaging to Measure Velocity Distributions in Surface Scattering Experiments. *J. Phys. Chem. A* **119**, 12255-12262, doi:10.1021/acs.jpca.5b06272 (2015).

6 Kresse, G. & Furthmüller, J. Efficient iterative schemes for *ab initio* total-energy calculations using a plane-wave basis set. *Phys. Rev. B* **54**, 11169-11186, doi:10.1103/PhysRevB.54.11169 (1996).

7 Kresse, G. & Furthmüller, J. Efficiency of *ab-initio* total energy calculations for metals and semiconductors using a plane-wave basis set. *Comput. Mater. Sci.* **6**, 15-50, doi:10.1016/0927-0256(96)00008-0 (1996).

8 Perdew, J. P., Burke, K. & Ernzerhof, M. Generalized Gradient Approximation Made Simple. *Phys. Rev. Lett.* **77**, 3865-3868, doi:10.1103/PhysRevLett.77.3865 (1996).

9 Blöchl, P. E. Projector augmented-wave method. *Phys. Rev. B* **50**, 17953-17979, doi:10.1103/PhysRevB.50.17953 (1994).

10 Zhang, Y., Hu, C. & Jiang, B. Embedded Atom Neural Network Potentials: Efficient and Accurate Machine Learning with a Physically Inspired Representation. *J. Phys. Chem. Lett.* **10**, 4962-4967, doi:10.1021/acs.jpclett.9b02037 (2019).

11 Matsumi, Y. *et al.* Doppler profiles and fine-structure branching ratios of $O(^3P_j)$ from photodissociation of carbon dioxide at 157 nm. *J. Chem. Phys.* **95**, 7311-7316, doi:10.1063/1.461408 (1991).

12 Stolow, A. & Lee, Y. T. Photodissociation dynamics of CO_2 at 157.6 nm by photofragment-translational spectroscopy. *J. Chem. Phys.* **98**, 2066-2076, doi:10.1063/1.464238 (1993).
